# Cathepsin K deficiency in mice induces structural and metabolic changes in the central nervous system that are associated with learning and memory deficits

**DOI:** 10.1186/1471-2202-12-74

**Published:** 2011-07-27

**Authors:** Stephanie Dauth, Ruxandra F Sîrbulescu, Silvia Jordans, Maren Rehders, Linda Avena, Julia Oswald, Alexander Lerchl, Paul Saftig, Klaudia Brix

**Affiliations:** 1 School of Engineering and Science, Research Center MOLIFE - Molecular Life Science, Jacobs University Bremen, Campus Ring 1, 28759 Bremen, Germany; 2 Institute of Biochemistry, Christian-Albrechts Universität Kiel, 24118 Kiel, Germany; 3 Department of Biology, Northeastern University, 360 Huntington Avenue, Boston, MA 02115, USA; 4 Silvia Jordans' current address is Institute of Biochemistry and Molecular Biology, Friedrich-Wilhelms Universität Bonn, 53115 Bonn, Germany

## Abstract

**Background:**

Cathepsin K is a cysteine peptidase known for its importance in osteoclast-mediated bone resorption. Inhibitors of cathepsin K are in clinical trials for treatment of osteoporosis. However, side effects of first generation inhibitors included altered levels of related cathepsins in peripheral organs and in the central nervous system (CNS). Cathepsin K has been recently detected in brain parenchyma and it has been linked to neurobehavioral disorders such as schizophrenia. Thus, the study of the functions that cathepsin K fulfils in the brain becomes highly relevant.

**Results:**

Cathepsin K messenger RNA was detectable in all brain regions of wild type (WT) mice. At the protein level, cathepsin K was detected by immunofluorescence microscopy in vesicles of neuronal and non-neuronal cells throughout the mouse brain. The hippocampus of WT mice exhibited the highest levels of cathepsin K activity in fluorogenic assays, while the cortex, striatum, and cerebellum revealed significantly lower enzymatic activities. At the molecular level, the proteolytic network of cysteine cathepsins was disrupted in the brain of cathepsin K-deficient (*Ctsk*^-/-^) animals. Specifically, cathepsin B and L protein and activity levels were altered, whereas cathepsin D remained largely unaffected. Cystatin C, an endogenous inhibitor of cysteine cathepsins, was elevated in the striatum and hippocampus, pointing to regional differences in the tissue response to *Ctsk *ablation. Decreased levels of astrocytic glial fibrillary acidic protein, fewer and less ramified profiles of astrocyte processes, differentially altered levels of oligodendrocytic cyclic nucleotide phosphodiesterase, as well as alterations in the patterning of neuronal cell layers were observed in the hippocampus of *Ctsk*^-/- ^mice. A number of molecular and cellular changes were detected in other brain regions, including the cortex, striatum/mesencephalon, and cerebellum. Moreover, an overall induction of the dopaminergic system was found in *Ctsk*^-/- ^animals which exhibited reduced anxiety levels as well as short- and long-term memory impairments in behavioral assessments.

**Conclusion:**

We conclude that deletion of the *Ctsk *gene can lead to deregulation of related proteases, resulting in a wide range of molecular and cellular changes in the CNS with severe consequences for tissue homeostasis. We propose that cathepsin K activity has an important impact on the development and maintenance of the CNS in mice.

## Background

Cathepsin K is a mammalian cysteine peptidase that is sorted to endo-lysosomes and secreted into the extracellular space by certain cell types [1]. It is abundant in osteoclasts and has a prominent role in bone remodeling due to its collagenolytic activity [2]. Indeed, cathepsin K deficiency has been linked to bone disorders such as pycnodysostosis and osteopetrosis [3,4], while the excessive activity of this enzyme may lead to osteoporosis [5], making it a likely target for rational drug design [6-8]. Cathepsin K has also been identified in a variety of cell types other than osteoclasts, such as macrophages [9], bronchial and alveolar epithelia [10], gastrointestinal mucosa [11], and thyroid epithelia [8,12,13], indicating that its distribution and functions may not be as clearly defined as initially thought. In the human brain, cathepsin K has been shown to be present in the choroid plexus [11], and recent studies have demonstrated its widespread distribution within neurons and glial cells [14].

To date, cathepsin K distribution and functions in the mouse CNS are largely unknown, however *Ctsk*^-/- ^mice have not been reported to suffer from severe neurobehavioral phenotypes [15]. The phenomenon of compensation, i.e. upregulation of related cysteine proteases, has been suggested to explain the mild phenotypes of mice with single deficiencies in cysteine cathepsins [1,16]. For example, enzyme compensation has been previously observed in the thyroid of *Ctsk*^-/- ^mice, where cathepsin L is significantly upregulated [16], as well as in the brain of *Ctsd*^-/- ^mice, where elevated levels of cathepsin B have been detected [17]. Moreover, the absence of cathepsins B and L is known to carry dramatic consequences, including neurodegeneration and brain atrophy [18,19], while the impact of cathepsin K deficiency on the mouse CNS has not been investigated in sufficient detail to date.

Interestingly, basic cathepsin K inhibitors previously tested in clinical trials have been reported to increase the activity levels of cathepsins B and L not only in the liver, kidney and spleen, but also in the CNS of rats [20], indicating that risk assessments of potential side effects of cathepsin inhibitor treatments must consider changes in the proteolytic network of both peripheral organs and the CNS [8]. Several pathways by which systemically reduced cathepsin K activity could cause alterations at the level of the brain are plausible: a direct effect on the metabolism of neurons and glial cells, via modulation of related enzymes, and/or indirectly, via yet unknown pathways.

In the present study, we used reverse transcription polymerase chain reaction (RT-PCR), immunolabeling, and enzyme histochemistry to demonstrate the expression of cathepsin K in the mouse brain, both at the mRNA and at the protein level. Moreover, cathepsin K activity assays revealed that specific enzyme activity in the hippocampus was higher than in other brain regions investigated. Therefore, we used *Ctsk*^-/- ^mice to investigate the systemic impact of cathepsin K gene ablation on the structure and metabolism of the brain. Immunoblotting and proteolytic activity assays were employed in WT and *Ctsk*^-/- ^mice to determine activity levels of related aspartic and cysteine cathepsins with an established function in the mouse CNS, and to assess the levels of a main endogenous cysteine cathepsin inhibitor, cystatin C. We further addressed the status of major brain regions including the cerebral cortex, the mesostriatal complex, the hippocampus, and the cerebellum, by using immunoblotting and immunohistochemistry to evaluate molecular markers of neuronal and glial cells. Potential behavioral alterations were analyzed in a series of tests chosen to monitor locomotor activity, anxiety levels, as well as spatial and non-spatial learning and memory.

Our results indicate that learning and memory impairments in *Ctsk*^-/- ^mice correlated with a combination of alterations in proteolytic pathways, including significant reductions of cathepsin B and L levels in cortex and striatum as well as increased cystatin C levels in striatum and hippocampus. In addition, some of the most striking observations included immature astrocytes in hippocampal regions of *Ctsk*^-/- ^animals, significant alterations in neuronal synaptotagmin levels, and prominent changes in the cyclic nucleotide phosphodiesterase (CNPase) levels of oligodendrocytes, indicating a major influence of cathepsin K on the cellular metabolism and function in the mouse CNS. In further support of this conclusion, a significantly altered distribution and patterning of neurons was observed throughout the hippocampus of *Ctsk*^-/- ^mice. We propose that the systemic disruption of cathepsin K activity during development and adulthood has severe local impacts on the structural integrity of the mouse CNS, eventually resulting in functional impairments such as learning and memory deficits.

## Results

### Cathepsin K identification in the mouse CNS

**Figure 1:**
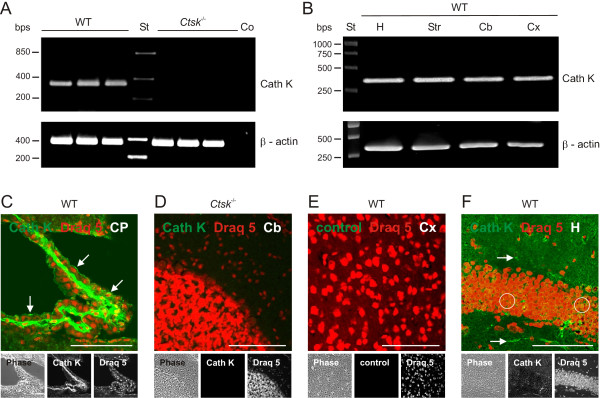
**Cathepsin K detection in mouse brain parenchyma**. Messenger RNA was isolated from tissue extracts prepared from different brain regions of WT or *Ctsk*^-/- ^mice. RT-PCR reactions with the primer pairs cathepsin K 5'-GCC AGG ATG AAA GTT GTA TG-3' (forward) and 5'-CAG GCG TTG TTC TTA TTC C-3' (reverse) as well as β-actin 5'-GCC AGG ATG AAA GTT GTA TG-3' (forward) and 5'-CAG GCG TTG TTC TTA TTC C-3' (reverse) resulted in the amplification of cDNAs of the expected lenghts of approximately 358 bps for cathepsin K specific mRNA (Cath K) and 335 bps for the loading control (β-actin) as revealed by agarose gel (1,5%) electrophoresis. In negative controls, cDNA template was omitted (Co). Markers of fragment sizes are given in lanes labeled with St. **(A) **Cathepsin K mRNA was detected by RT-PCR in cerebral cortex tissue from WT mice (n = 3), but not in samples obtained from *Ctsk*^-/- ^mice (n = 3; upper panel). **(B) **Cathepsin K mRNA was detected in all four investigated brain regions in WT animals. Bps - base pairs; St - standard DNA ladder; Co - negative RT-PCR control without template; H - hippocampus; Str - striatum/mesencephalon; Cb - cerebellum; Cx - cortex. Confocal laser scanning micrographs were taken from horizontal sections through choroid plexus (**C**), cerebellum (**D**), cortex (**E**), and hippocampus (**F**) of WT or *Ctsk*^-/- ^mice as indicated. Sections were prepared from PFA-fixed brain tissue, immunostained for cathepsin K (green signals, lower panels - middle), counter-stained for nuclear DNA with Draq 5 (red signals, lower panels - right), and inspected with fluorescence and phase contrast (lower panels - left) microscopy. Cathepsin K protein was immunodetected in ependymal cells (**C**, arrows), neurons (**F**, circles) and glial cells (**F**, arrows) of WT mice (C and F) but not in negative antibody controls (**E**) or in sections prepared from *Ctsk*^-/- ^brain tissue (**D**), indicating specificity of cathepsin K immunolabeling. Bars - 100 μm **(C-F)**. CP - choroid plexus; Cb - cerebellum; Cx - cortex; H - hippocampus.

Several studies have described the presence of cathepsin K in the CNS of rats [21,22] and in *post-mortem *human brain tissue [14,23]. In order to determine whether cathepsin K is also present in the mouse CNS, tissue extracts were prepared from cerebral cortex of WT and *Ctsk*^-/- ^mice and examined by RT-PCR. Cathepsin K mRNA expression was detectable in the cerebral cortex of WT animals but it was absent in *Ctsk*^-/- ^mice (Figure 1A). Cathepsin K mRNA was also present in extracts prepared from other brain regions, such as hippocampus, striatum/mesencephalon, and cerebellum of WT mice (Figure 1B), suggesting a broad distribution of cathepsin K throughout the mouse CNS.

**Figure 2:**
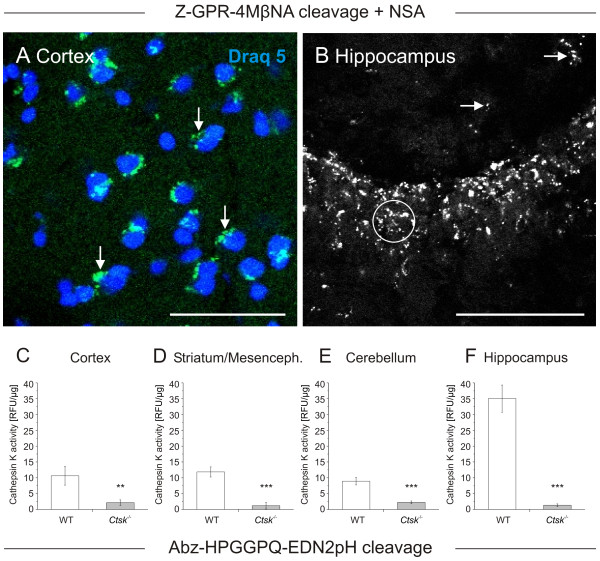
**Visualization and quantification of cathepsin K activity in the CNS of WT mice**. Confocal laser scanning micrographs were taken from horizontal sections through cortex (**A**) and hippocampus (**B**) of WT mice as indicated. Sections were prepared from 0.5% PFA-fixed brain tissue after a 45-min reaction with the cathepsin K substrate Z-Gly-Pro-Arg-4MβNA in the presence of NSA as precipitating agent of released 4MβNA under oxygen-free conditions and at pH 6.2 (**A**, green signals, and **B**, white signals), and after counter-staining with Draq 5 (**A**, blue signals). Cathepsin K activity was detected in vesicles (**A**, arrows) of both neuronal (**B**, circle) and glial cells (**B**, arrows) in the different brain regions analyzed by enzyme histochemistry. Bars - 100 μm (**A**), 50 μm (**B**). Results of cathepsin K activity assays performed with brain tissue lysates of WT (**C-F**, open bars, n = 6) and *Ctsk*^-/- ^mice (grey bars, n = 3). Cathepsin K activity as determined by cleavage of Abz-HPGGPQ-EDN_2_ph at pH 5.5 was detected in the cortex, striatum/mesencephalon, cerebellum, and hippocampus of WT animals. Specific cathepsin K activity levels were calculated by division of relative fluorescence units (RFU) by the protein content of the respective samples. Specificity of the assay was proven by the use of tissue from cathepsin K deficient mice. Cathepsin K activity was well detectable in all brain regions analyzed and was highest in the hippocampus (**C-F**, open bars). Mean values ± standard deviations are depicted; levels of significance are denoted as ** for p < 0.01; *** for p < 0.001.

Indirect immunofluorescence studies revealed cathepsin K being translated in various brain regions of WT mice but not in *Ctsk*^-/- ^animals (Figure 1C and 1F, cf. 1D, respectively). Moreover, cathepsin K activity was detectable in vesicles of neurons and glial cells by enzyme histochemistry [12] performed on cryo-sections prepared from WT mouse brains (Figure 2A and 2B, arrows). In particular, the CA2 region of the hippocampus exhibited signals derived from cathepsin K substrate conversion both in stratum pyramidale (Figure 2B, circle) as well as in stratum radiatum (arrows).

In order to further verify the specificity of the observed immunofluorescence and cathepsin K substrate conversion reactions, brain tissue homogenates prepared from WT and *Ctsk*^-/- ^mice were used to perform cathepsin K activity assays, employing Abz-HPGGPQ-EDN_2_pH as a fluorogenic substrate [24] (Figure 2C - 2F). Cathepsin K activity was detected in all analyzed brain regions of WT mice (Figure 2C - 2F, open bars), whereas no activity was detectable in extracts from brain tissue of *Ctsk*^-/- ^animals (grey bars). Importantly, specific cathepsin K activity reached highest levels in the hippocampus, while the cortex, striatum/mesencephalon, and the cerebellum exhibited significantly lower cathepsin K activity in WT mice (Figure 2C - 2F, open bars).

These results indicated cathepsin K expression and suggested its enzymatic functionality throughout the mouse CNS. Hence, we analyzed the impact of cathepsin K deficiency on different regions of the mouse brain at the molecular and cellular level.

### Impact of cathepsin K deficiency on the proteolytic network of related cathepsins in the mouse CNS

It is known that cathepsin networks are able to compensate for the loss of function of individual enzymes. If the activity of a given cathepsin is inhibited, either chemically or through gene ablation, related cathepsins might be upregulated in a cell type- and tissue-specific manner. In order to investigate such potential network effects induced by the loss of cathepsin K, the protein and activity levels of cathepsins B, D, and L and the protein levels of the endogenous cysteine peptidase inhibitor cystatin C were determined by immunoblotting and proteolytic activity assays in tissue extracts prepared from different regions of the CNS of WT and *Ctsk*^-/- ^mice.

Cathepsin D protein levels were not altered significantly in any of the investigated brain regions (Additional file 1 and Additional file 2). These results suggested that the aspartic cathepsin D is not affected by cathepsin K deficiency. By contrast, protein levels of cathepsins B (p < 0.001) and L (p < 0.05) (Additional file 1 and Additional file 2) were significantly reduced in cerebral cortex of *Ctsk*^-/- ^compared to WT mice, although no significant differences were observed in the activity levels. The expression levels of the endogenous inhibitor cystatin C were not altered (Additional file 1). In the striatum/mesencephalon a significant decrease in the enzymatic activity of both cathepsins B (p < 0.001) and L (p < 0.05) was observed while cystatin C levels were increased (p < 0.05) in this brain region (Additional file 1). Cathepsin B protein and activity levels remained unaffected in the cerebellum and hippocampus of *Ctsk*^-/- ^mice (Additional file 1). Cathepsin L activity levels were slightly but not significantly reduced in cerebellum and hippocampus, although cathepsin L protein levels were increased in the hippocampus (p < 0.05; Additional file 1). As in the striatum/mesencephalon, an increase of cystatin C protein levels (p < 0.001) was observed in the hippocampus of *Ctsk*^-/- ^when compared to WT mice (Additional file 1). Overall, these results indicate a deregulated proteolytic network within various regions of the *Ctsk*^-/- ^mouse brain, affecting in particular cysteine cathepsins.

### Impact of cathepsin K deficiency on neuronal markers and cytoarchitecture in the hippocampus

**Figure 3:**
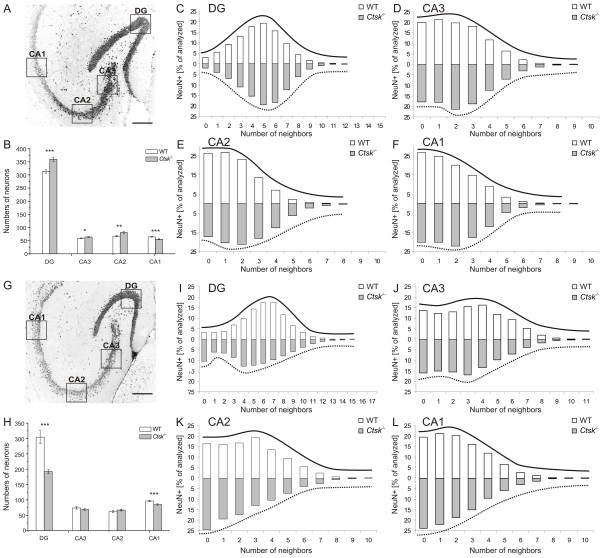
**Distribution and numbers of neurons in the hippocampus**. Confocal laser scanning micrographs were taken from serial horizontal sections through the dorsal **(A) **and the ventral hippocampus **(G) **that were immunostained for NeuN to detect the nuclei of neuronal cells. To quantify cytoarchitectural variations in the structure of neuronal cell layers, the numbers of neighbours for any given cell were determined as a measure of density of cell packing. In the dorsal hippocampus of WT animals (n = 6), fewer cells were surrounded by a high number of neighbours, i.e. neurons showed a more sparse pattern **(C-F)**. By contrast, in *Ctsk*^-/- ^mice (n = 6) the ventral part of the hippocampus showed a significantly more sparse neuronal distribution **(G-L)**. This patterning difference was most pronounced in the DG **(C and I)**, CA2 **(E and K) **and CA1 **(F and L)**, although all regions exhibited significant differences in the patterning of the neuronal layer between the two genotypes (p < 0.001, Kolmogorov-Smirnov test). **(B) **Numbers of neurons were increased in DG, CA3 and CA2 regions in the dorsal part of the hippocampus of *Ctsk*^-/- ^compared to WT mice, whereas neuronal numbers were decreased in the CA1 region of *Ctsk*^-/- ^mice. **(H) **Fewer neurons were observed in DG and CA1 region in the ventral area of the hippocampus of *Ctsk*^-/- ^mice. Bars - 200 μm **(A, G)**. Fluorescence micrographs are displayed in reverse contrast.

Deregulated cysteine cathepsins and altered cystatin B/stefin B and/or cystatin C levels are known to negatively influence the cellular architecture of the mouse CNS. Both neurons and glia are affected in a number of mouse models with deficiencies in cysteine cathepsins and their endogenous inhibitors, i.e. cystatins B and C, resulting in dramatic loss of vital brain functions [18,19,25]. Since cathepsin K activity was highest in the hippocampus and because an impairment of this brain structure, which is involved in memory formation and spatial mapping [26], could explain the learning deficits observed in *Ctsk*^-/- ^mice, we further examined its neuronal patterning. Differentiating between the dorsal and ventral hippocampus, we assessed the impact of cathepsin K deficiency by examining the specific cell patterning, i.e. the numbers and densities of neurons in the dentate gyrus (DG), CA1, CA2, and CA3 regions (Figure 3).

Immunohistochemical identification of NeuN-positive neuronal nuclei in serial cryo-sections and semi-automated pattern analyses revealed significant differences in the overall distribution of neurons in all investigated areas in the dorsal and ventral hippocampus of *Ctsk*^-/- ^mice in comparison to WT (p < 0.001, two-sample Kolmogorov-Smirnov test). Neurons were identified and counted using image analysis software (CellProfiler 2.0, version 9777). In order to analyze neuronal patterning, the number of neighbours per neuron within a radius of 7 μm or lower was initially determined. Patterning was interpreted as dense when neurons were surrounded by many neighbours, while a sparse patterning was indicated by a predominance of neurons surrounded by few neighbours. For instance, typical patterning in WT mice in the DG was characterized by most neurons having 5 neighbours in dorsal versus 6-7 neighbours in ventral hippocampus (Figure 3C and 3I, respectively, white bars).

The dorsal DG of *Ctsk*^-/- ^mice showed a more densely packed distribution of neurons, with numerous neurons surrounded by many neighbours, whereas the opposite was true for WT mice, in which most neurons had relatively few neighbours (Figure 3C). This different patterning in *Ctsk*^-/- ^mice was most obvious when comparing the shifts in the maxima of bell-shaped curves profiling the numbers of neuronal neighbours to those in WT animals (Figure 3, straight lines profiling white bars versus dotted lines profiling grey bars). This difference - more densely packed neurons in *Ctsk*^-/- ^mice - was observed in all investigated areas of the dorsal hippocampus (Figure 3C - 3F). Counting also revealed a significant increase in the numbers of NeuN-positive cells in the dorsal DG, CA3, and CA2 regions of *Ctsk*^-/- ^compared to WT mice (Figure 3B; p < 0.001; p < 0.05 and p < 0.001, respectively). A slight, but significant reduction of NeuN-positive cell nuclei was observed in the dorsal CA1 region of *Ctsk*^-/- ^mice (Figure 3B; p < 0.001).

In contrast to the dorsal part, the ventral hippocampus showed strikingly different alterations in neuronal numbers and distribution in consequence of cathepsin K deficiency. WT animals exhibited more densely packed neurons in the ventral hippocampus, whereas *Ctsk*^-/- ^mice had more neurons with fewer neighbours in all investigated areas. This is indicative of increased neuronal spreading in the ventral hippocampus of *Ctsk*^-/- ^animals (Figure 3I - 3L). These alterations were most obvious in the DG, although the overall neuronal patterning was significantly different in all areas of the ventral hippocampus between the two genotypes. Furthermore, the numbers of NeuN-positive cells in the ventral DG and CA1 regions of *Ctsk*^-/- ^mice were significantly decreased as compared to WT controls (Figure 3H; p < 0.001).

Overall, these results suggest subtle changes in the cytoarchitecture of the hippocampus of *Ctsk*^-/- ^mice, with a gain of neurons in its dorsal part at the expense of a loss of neurons in its ventral part. Furthermore, a denser packing of neurons was observed in dorsal hippocampal regions, whereas neurons had a sparser distribution in the ventral hippocampus of *Ctsk*^-/- ^mice.

**Figure 4:**
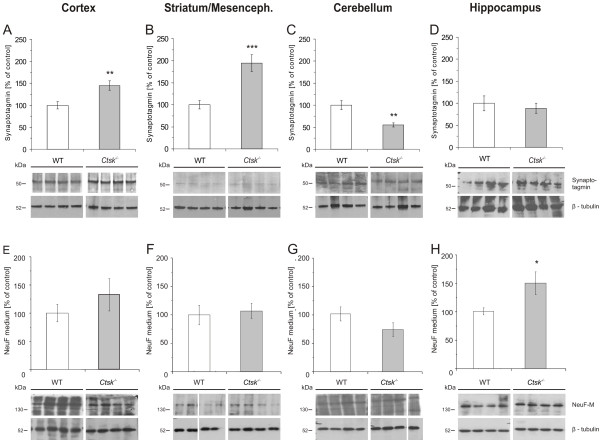
**Neuronal markers in the cerebral cortex, striatum/mesencephalon, hippocampus, and cerebellum**. **(A, E) **Densitometry analysis of immunoblots prepared from lysates of cerebral cortex of WT (white bars) and *Ctsk*^-/- ^mice (grey bars) as indicated. Synaptotagmin levels were significantly elevated by approximately 45% in *Ctsk*^-/- ^mice as compared to WT (A, *Ctsk*^-/- ^n = 10; WT n = 13), while analysis of the same samples showed no significant difference in the amount of NF-M protein (E, *Ctsk*^-/- ^n = 8; WT n = 6). **(B, F) **Densitometry analysis of immunoblots prepared from lysates of striatum/mesencephalon of WT (white bars) and *Ctsk*^-/- ^mice (grey bars) as indicated. Synaptotagmin levels were elevated in *Ctsk*^-/- ^mice by 95% as compared to WT mice (B, *Ctsk*^-/- ^n = 10; WT n = 10), while no significant difference was observed in NF-M protein (F, *Ctsk*^-/- ^n = 9; WT n = 11). **(C, G) **Densitometry analysis of immunoblots prepared from lysates of cerebellum of WT (white bars) and *Ctsk*^-/- ^mice (grey bars) as indicated. Synaptotagmin levels were significantly down-regulated in *Ctsk*^-/- ^mice by approximately 55% compared to WT animals (C, *Ctsk*^-/- ^n = 10; WT n = 13), while no significant difference was observed in NF-M protein between the two genotypes (G, *Ctsk*^-/- ^n = 9; WT n = 10). **(D, H) **Densitometry analysis of immunoblots prepared from lysates of hippocampus of WT (white bars) and *Ctsk*^-/- ^mice (grey bars) as indicated. No significant difference was detected in the amount of synaptotagmin in *Ctsk*^-/- ^mice compared to WT (D, *Ctsk*^-/- ^n = 10; WT n = 13), while NF-M protein was elevated by approximately 50% in *Ctsk*^-/- ^mice compared to WT (H, *Ctsk*^-/- ^n = 11; WT n = 9). Protein levels were determined by densitometry and grey values per area were normalized to the loading control β-tubulin. Bar charts denote mean values expressed as percent of control ± standard error; levels of significance are denoted as * for p < 0.05; ** for p < 0.01; *** for p < 0.001. Representative immunoblots are shown in the lower panels; lanes represent separate individuals.

In order to detect other potential abnormalities in neuronal distribution and function, immunoblotting experiments were performed to assess CNS levels of the pre-synaptic marker synaptotagmin and of the intermediate filament protein NF-M. Densitometry analysis of immunoblots revealed a significant increase in synaptotagmin levels in the striatum/mesencephalon of almost 100% (p < 0.001; Figure 4B), and of approximately 45% (p < 0.01; Figure 4A) in the cerebral cortex of *Ctsk*^-/- ^mice compared to WT controls. By contrast, there was a significant decrease in synaptotagmin amounts in the cerebellum (p < 0.01; Figure 4C) of *Ctsk*^-/- ^mice, while hippocampal synaptotagmin levels remained unaltered (Figure 4D). For NF-M protein, the results demonstrated a 50% increase (p < 0.05) in the hippocampus of *Ctsk*^-^^/- ^mice in comparison to WT controls (Figure 4H).

### Impact of cathepsin K deficiency on glial cells

**Figure 5:**
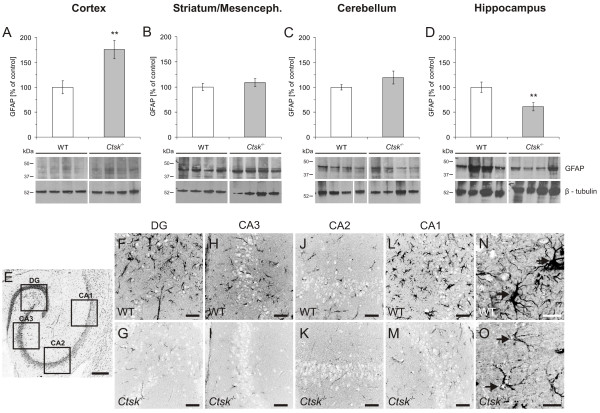
**GFAP status in cerebral cortex, striatum/mesencephalon, cerebellum, and hippocampus**. **(A-D) **Densitometry analysis of immunoblots prepared from lysates of cerebral cortex, striatum/mesencephalon, cerebellum and hippocampus of WT (white bars) and *Ctsk*^-/- ^mice (grey bars) as indicated. Representative immunoblots are shown in the lower panels; lanes represent separate individuals. **(A) **GFAP levels in the cerebral cortex showed a significant increase of approximately 76% in *Ctsk*^-/- ^mice compared to WT (A, *Ctsk*^-/- ^n = 10; WT n = 13). **(B) **No significant difference was observed in the amount of GFAP in striatum/mesencephalon (B, *Ctsk*^-/- ^n = 10; WT n = 10). **(C) **No alterations were observed in GFAP levels in cerebellum (C, *Ctsk*^-/- ^n = 10; WT n = 12). **(D) **GFAP levels were significantly decreased in hippocampus of *Ctsk*^-/- ^mice, reaching approximately 60% of the values observed in WT (D, *Ctsk*^-/- ^n = 10; WT n = 10). **(E-O) **Developmental status of astrocytes in the hippocampus. **(E) **Overview of a horizontal section through the hippocampus that was immunostained for GFAP to detect mature astrocytes, illustrating the analyzed regions. **(F-O) **Confocal laser scanning micrographs of sections immunolabeled against GFAP showed strongly decreased staining intensity in samples from *Ctsk*^-/- ^mice as compared to WT. The same pattern could be observed in the dentate gyrus **(F, G)**, CA3 region **(H, I)**, CA2 region **(J, K)**, and CA1 region **(L, M)**. **(N, O) **Higher magnification images of astrocytes in the CA1 area. In contrast to the dense astrocytic profiles observed in WT, those of *Ctsk*^-/- ^mice appeared much thinner and less ramified (arrows). Bars - 200 μm in **(E)**, 50 μm in **(F-O)**. Fluorescence micrographs are displayed in reverse contrast. Levels of significance are denoted as ** for p < 0.01.

In order to further assess the structural changes induced by the ablation of cathepsin K, we investigated the status of the astroglia and oligodendrocytes in the CNS of *Ctsk*^-/- ^mice. We observed a significant increase of approximately 76% in the expression of the astrocyte marker glial fibrillary acidic protein (GFAP) in the cerebral cortex of *Ctsk*^-/- ^mice (p < 0.01, Figure 5A) and a decrease of 40% in the hippocampus (p < 0.01, Figure 5D) as compared to WT animals. To investigate whether the marked decrease in GFAP expression might be connected to the differentiation state of astrocytes in the hippocampus of *Ctsk*^-/- ^mice, immunohistochemistry was used to identify GFAP in tissue sections (Figure 5E - 5O). In all main areas of the hippocampus, including DG, CA3, CA2, and CA1 areas, reduced GFAP immunolabeling was observed in *Ctsk*^-/- ^animals as compared to WT controls (Figure 5F - 5M). Detailed observation of individual astrocytes in the CA1 region also revealed less numerous and apparently thinner ramifications of *Ctsk*^-/- ^mice astroglia (Figure 5N and 5O). These results indicated that the prevalence of underdeveloped astrocytes might underlie the quantified decrease in GFAP levels.

**Figure 6:**
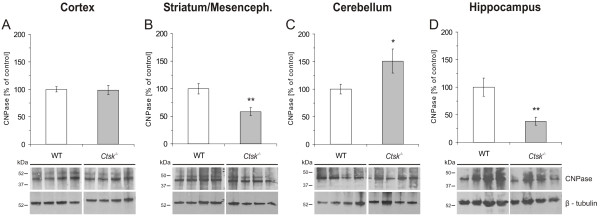
**CNPase status in cerebral cortex, striatum/mesencephalon, cerebellum, and hippocampus**. **(A-D) **Densitometry analysis of immunoblots prepared from lysates of cerebral cortex, striatum/mesencephalon, cerebellum, and hippocampus of WT (white bars) and *Ctsk*^-/- ^mice (grey bars) as indicated. Representative immunoblots are shown in the lower panels; lanes represent separate individuals. **(A) **CNPase levels showed no significant difference in cerebral cortex of *Ctsk*^-/- ^mice compared to WT controls (A, *Ctsk*^-/- ^n = 10; WT n = 13). **(B) **In striatum/mesencephalon CNPase levels were significantly lower in *Ctsk*^-/- ^mice, reaching only 58% of values measured in WT (B, *Ctsk*^-/- ^n = 10; WT n = 10). **(C) **In cerebellum CNPase levels were significantly increased in *Ctsk*^-/- ^mice by 50% as compared to WT (C, *Ctsk*^-/- ^n = 10; WT n = 11). **(D) **CNPase levels in hippocampus were significantly decreased in *Ctsk*^-/- ^mice, reaching only 40% of values measured in WT controls (D, *Ctsk*^-/- ^n = 11; WT n = 11). Levels of significance are denoted as * for p < 0.05; ** for p < 0.01.

Densitometry analysis of immunoblots labeled for CNPase, an oligodendrocyte marker, revealed a significant decrease of approximately 42% in CNPase levels in the striatum/mesencephalon (p < 0.01; Figure 6B) as well as a decrease of approximately 60% in the hippocampus of *Ctsk*^-/- ^mice (p < 0.01; Figure 6D). In the cerebellum, however, CNPase levels were increased by approximately 50% in *Ctsk*^-/- ^mice (p < 0.05; Figure 6C).

**Figure 7:**
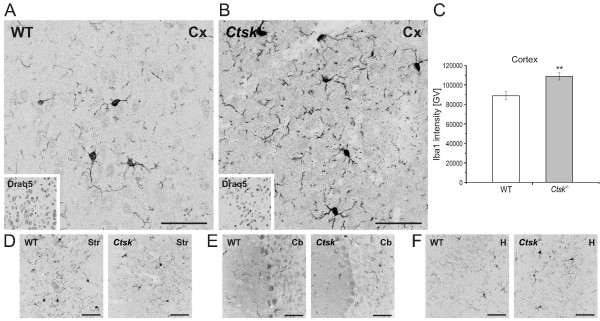
**Status of microglia in *Ctsk*^-/- ^and WT mice**. Representative confocal laser scanning micrographs of horizontal sections of the cerebral cortex immunolabeled against Iba1 to detect microglia revealed a pronouncedly increased staining in *Ctsk*^-/- ^mice as compared to WT **(A, B)**. **(C) **Fluorescence intensity analysis of sections immunolabeled for Iba1 indicated a significant increase in Iba1 signal in *Ctsk*^-/- ^mice in the cerebral cortex as compared to WT mice (n = 10 per genotype). Fluorescence intensities were determined as grey value (GV) sums over the entire image, and are given as means ± standard error. **(D-F) **Representative confocal micrographs of sections immunolabeled against Iba1 revealed that no significant differences were detectable in striatum/mesencephalon, cerebellum or hippocampus of *Ctsk*^-/- ^animals in comparison to WT. Cx - cerebral cortex; Str - striatum/mesencephalon; Cb - cerebellum; H - hippocampus. Bars - 50 μm. Fluorescence micrographs are displayed in reverse contrast. Levels of significance are denoted as ** for p < 0.01.

Since cathepsin K has already been reported to be involved in the immune response [27], we further analysed the state of microglia, the resident macrophages of the CNS [28]. Immunohistochemistry analysis of brain sections labeled with ionized calcium binding adapter protein-1 (Iba1), a marker of microglia, showed a slightly stronger signal in the cerebral cortex of *Ctsk*^-/- ^mice compared to WT controls (p < 0.01; Figure 7A - 7C), whereas no alterations in the Iba1 signal were detectable in striatum/mesencephalon, cerebellum, or hippocampus (Figure 7D - 7F).

### Behavioral effects of cathepsin K deficiency

**Figure 8:**
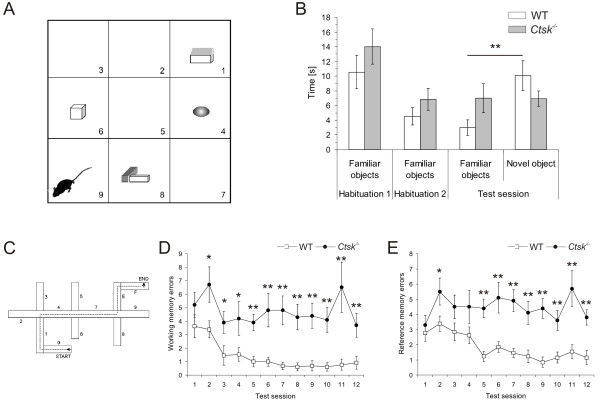
**Learning and memory abilities of *Ctsk*^-/- ^mice compared to WT controls analyzed by a novel object recognition test and by an elevated multiple choice maze**.**(A) **Experimental setting of the novel object recognition test. Individual mice were placed in the lower left square, facing the corner. After two habituation sessions, the cube in square 6 was replaced with a shell (objects not drawn to scale). **(B) **Both WT (n = 13) and *Ctsk*^-/- ^mice (n = 10) showed, after habituation, a marked decrease in the time spent in any object's proximity (left). During the test session, the WT mice spent significantly more time exploring the novel object than they spent with any of the familiar objects (right, open bars). *Ctsk*^-/- ^mice (grey bars) did not show any preference for the new object over the familiar ones. **(C) **Experimental setting of the elevated multiple choice maze. Animals were placed in the starting segment of the maze, and had to make three correct decisions in order to reach the exit point, where they could descend via a wire net into a cage containing a house. The cage was placed on the floor and was not readily visible from the maze. Testing was conducted daily, over an interval of 12 days. An entry into a maze segment that was outside the correct route (0-1-4-7-E-F in this order) was counted as a reference memory error, while backtracking into any already visited segment within a given session was counted as a working memory error. **(D) **The learning curves for the two groups were compared on each day of the experiment. *Ctsk*^-/- ^mice made significantly more working memory errors already from day 2 of testing, in contrast to WT controls (B, *Ctsk*^-/- ^n = 10; WT n = 13) which showed a normal learning curve. **(E) **Similarly, *Ctsk*^-/- ^mice made significantly more reference memory errors starting from day 5 of testing (C, *Ctsk*^-/- ^n = 10; WT n = 13). Levels of significance are denoted as * for p < 0.05; ** for p < 0.01.

The differences in the expression of molecular factors, particularly those affecting the dopaminergic system (see below) and the hippocampus (see above), were paralleled by observed alterations in the behavior of *Ctsk*^-/- ^mice. The dopaminergic system has been associated with a wide range of cognitive processes, such as learning, memory, novelty seeking, reward systems, but also motor regulation of movement [29]. The hippocampus is well established as vital for learning and memory as well as emotion-related behavior [30]. Here, we have tested *Ctsk*^-/- ^mice on several measures assessing locomotor function and activity throughout the day (Additional file 3), anxiety (Additional file 4), as well as learning and memory (Figure 8).

Diurnal variations of in-cage activities of 5 animals per each genotype were determined over a time period of 3 weeks by a motion-triggered infrared sensor module. No significant alterations in frequencies of day or night activities were detectable (Additional file 3), arguing against any overt deficits in locomotor function of *Ctsk*^-/- ^mice up to an age of 6 months. In an elevated plus maze task *Ctsk*^-/- ^mice spent approximately 36% (108s out of 300s) in the open arms, whereas WT mice spent only about 19% (57s out of 300s) of total time in this area. Correspondingly, while the WT mice showed a pronounced preference for the closed arms of the maze (p < 0.001), in *Ctsk*^-/- ^mice this difference was not detectable (Additional file 4). Moreover, a hole board test revealed a significant increase in the frequency of central area crossing in *Ctsk*^-/- ^mice, supporting the hypothesis of reduced anxiety in this genotype (Additional file 4).

In order to test long-term non-spatial memory, a novel object recognition test was used. This assessment included two habituation sessions and a test session, when a familiar object was replaced with a novel one. During the final test session of the novel object recognition test WT mice spent significantly (p < 0.01) more time exploring the novel object than they spent with any of the familiar objects (Figure 8A and 8B, white bars). By contrast, *Ctsk*^-/- ^mice did not show any preference for the new object over the familiar ones, indicating that they could not discriminate the novel element placed in their familiar environment (Figure 8A and 8B, grey bars). Interestingly, during all sessions *Ctsk*^-/- ^mice made overall more direct contacts with all objects as compared to WT animals (data not shown), again indicating decreased inhibition levels.

To further investigate a potential learning deficit of *Ctsk*^-/- ^mice, an elevated multiple choice maze was used (Figure 8C), which allowed the testing of both short-term (working memory, WM) and long-term learning and memory proficiency (reference memory, RM). A repeated measures ANOVA, with test day as 'within subjects factor' and genotype as 'between subjects factor', indicated that *Ctsk*^-/- ^mice made significantly more reference memory errors (F(1,21) = 46.981, p < 0.0001) as well as working memory errors (F(1,21) = 60.273, p < 0.0001), as compared to WT mice (Figure 8D and 8E). As illustrated in Figures 8D and 8E, subsequent pairwise comparisons indicated a clear separation in performance levels for the two genotypes starting from day two (WM) or day five (RM). While WT mice displayed a normal learning curve with progressively fewer errors, *Ctsk*^-/- ^mice showed almost no improvement over the duration of the experiment.

### Cathepsin K and the dopaminergic system

**Figure 9:**
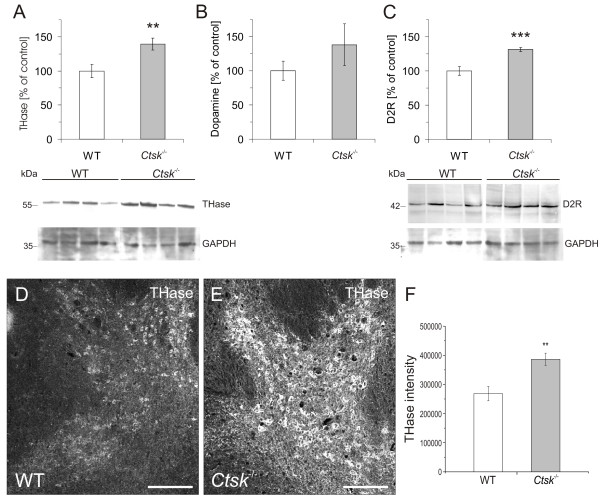
**Dopaminergic system markers in the striatum and mesencephalon**. **(A) **Immunoblot analysis of striatum/mesencephalon samples revealed a significant overall increase in the amount of tyrosine hydroxylase (THase) in *Ctsk*^-/- ^mice (n = 10) as compared to WT controls (n = 13). A representative immunoblot is shown in the lower panel; lanes represent separate individuals. **(B) **ELISA results from the same tissue lysates showed an average increase of 40% in the amount of dopamine in *Ctsk*^-/- ^mice. **(C) **Immunoblot analysis further indicated that levels of dopamine type 2 receptors (D2R) were significantly increased by approximately 30% in *Ctsk*^-^^/- ^mice. **(D, E) **THase immunolabeling of the VTA. At identical intensity gain settings, confocal fluorescence microscopy on horizontal brain sections labeled with an antibody against THase, resulted in an intense specific staining of the VTA of *Ctsk*^-/- ^mice, as compared to the moderate signal observed in the same region of WT brain sections. **(F) **Fluorescence intensity analysis of THase immunolabeled brain sections revealed a significant increase in THase intensity in *Ctsk*^-/- ^compared to WT mice. Bars - 200 μm. Levels of significance are denoted as ** for p < 0.01; *** for p < 0.001.

The dopaminergic system was investigated due to its widespread regulatory functions in movement and motor learning, novelty seeking, and reward processing. By pooling the striatum and mesencephalon we aimed to isolate the majority of dopaminergic neurons that form the mesostriatal pathways [31]. This allowed us to assess the influence of cathepsin K deficiency on the status of the dopaminergic system. Interestingly, densitometry analysis of immunoblots labeled for tyrosine hydroxylase (THase), a marker of catecholaminergic neurons, revealed a significant 39% overall increase (p < 0.01) in the amount of enzyme in the *Ctsk*^-/- ^mice (Figure 9A). To determine whether the increased levels of THase may lead to a variation in the levels of dopamine, an ELISA was performed on striatum/mesencephalon tissue lysates of WT and *Ctsk*^-/- ^mice (Figure 9B). Results showed an average increase of 40% in the amount of dopamine in *Ctsk*^-/- ^mice, however, this ascending trend did not reach statistical significance. To rule out that this trend towards higher dopamine levels in *Ctsk*^-/- ^mice might be mediated by decreased receptor levels, the blotted membranes were probed with antibodies specific for D2 receptors. Interestingly, results revealed a significant 30% upregulation (p < 0.001) of D2 receptors in *Ctsk*^-/- ^animals in comparison to WT (Figure 9C). To further explore the apparent induction of the dopaminergic system in *Ctsk*^-/- ^mice, we used immunolabeling for THase on brain tissue sections (Figure 9D and 9E). Confocal fluorescence microscopy on labeled brain sections revealed an intense staining of the ventral tegmental area (VTA) of *Ctsk*^-/- ^mice (Figure 9E), whereas less pronounced immunopositive signals were observed in the VTA of WT animals (Figure 9D). Intensity analysis of images of the THase immunolabeled sections, out of which representative examples are shown (Figure 9D and 9E), revealed a significant increase in staining intensity for THase in *Ctsk*^-/- ^mice compared to WT controls (Figure 9F).

## Discussion

Until recently, the expression of cathepsin K was believed to be limited to osteoclasts, where it serves a central function for the proper turnover of long bones [2-4]. Cathepsin K expression has since been shown in a variety of organs and tissues, and has been connected to numerous other functions, such as proper signalling in the context of the immune response [27] or the processing of β-endorphin in the brain [23]. Cathepsin K has also been associated with diseases, such as breast cancer [32] and schizophrenia [14]. Further functions of cathepsin K with a widespread impact on physiology and pathophysiology comprise its contributions in glucose metabolism [33] and in the processing of one of its natural substrates, thyroglobulin, thereby mediating liberation of thyroid hormones [8,12,13,16]. Hence, more detailed investigations concerning other potential roles of this protease are essential, especially considering the entry of cathepsin K inhibitors into clinical treatment of osteoporosis [7,8,34]. The present study aimed to characterize possible functions of cathepsin K in the mouse CNS. Our results demonstrate that cathepsin K is of considerably higher importance for the development, structure, and function of the CNS than previously thought.

From the results of this study, we concluded that the metabolism and structure of non-neuronal cells were significantly perturbed in the CNS of *Ctsk*^-/- ^animals. The numbers and the maturation level of astrocytes were decreased in the hippocampus, while oligodendrocyte markers were altered in all analyzed brain regions except the cortex. The distribution of microglia was modified only in the cortex. The analysis of neuronal markers demonstrated that the architecture of the neuronal layers was affected by cathepsin K deficiency in particular in the hippocampus, a region of the CNS known for its importance in the regulation of anxiety and memory. In line with this notion, we observed the highest specific activity of cathepsin K within this brain region.

Finally, behavioral studies showed that general locomotor function was not obviously affected in *Ctsk*^-/- ^mice, at least up to the age of 6 months. However, importantly, a clear impact of cathepsin K deficiency on learning and memory as well as novelty seeking was revealed.

### Linking brain and thyroid functions of cathepsin K

Previously, we have shown that cathepsin K- and L-double deficient mice are hypothyroid [16]. Hypothyroid conditions in early post-natal stages are considered to negatively affect the development of the CNS [35]. The abnormal state of cerebellar development during this period is one of the most obvious indications for severe hypothyroidism expected to affect adult brain structure and function significantly [36]. However, cerebellar development of *Ctsk*^*-/- *^mice at post-natal day 12 was indistinguishable from that of WT controls inspected at the same age. Moreover, thyroxine levels in the blood of the *Ctsk*^-/- ^animals analyzed in our previous study and those investigated in this study were not significantly altered in comparison to WT controls. Thus, although we initially hypothesized that the brain phenotype described in this study could be directly attributed to a mild hypothyroidism, we concluded that this hypothesis was supported neither by the status of early post-natal development of *Ctsk*^*-/- *^mice nor by the thyroid hormone levels determined in the brain of these animals (Sîrbulescu, Dauth, Rehders, Saftig, Jordans, Brix, unpublished). Therefore, we addressed other mechanisms through which cathepsin K deficiency could have affected brain development. Because we show herein that cathepsin K is indeed expressed in the mouse brain and exhibits proteolytic activity in particular in the hippocampus, we suggest a more direct effect of cathepsin K deficiency on the mouse CNS development. To unequivocally distinguish between the effects exerted by a lack of cathepsin K expression in either the thyroid or the CNS of mice, organ-specific knock-outs are required and planned for in our future investigations.

### Proteolytic network of the cathepsin K-deficient mouse CNS

Another pathway which might lead to the pronounced changes observed in the CNS of *Ctsk*^-/- ^mice is via modulation of related proteases. Compensatory phenomena underlying the mild phenotypes in mice lacking only one cysteine cathepsin have been previously described to occur in a cell type and tissue specific manner [1,8]. Interestingly, while cathepsins B and L, which are closely related to cathepsin K, showed altered expression levels in various brain regions, the distantly related aspartic protease cathepsin D was not affected in the brain of *Ctsk*^-/- ^animals.

Cathepsin B has been associated with neuronal survival by a variety of studies, often with conflicting results. While cathepsin B of microglial origin has been shown to serve as an initiator of apoptosis in cultures of hippocampal [25] and cortical neurons [37], its absence has been associated with the death of motor neurons in amyotrophic lateral sclerosis [38]. A cathepsin B-like protease has been implicated in dendritic spine collapse in hippocampal neurons, a process which mediates neuronal synaptic plasticity [39]. A decrease in protein level or activity of this protease, as observed in the cortex and striatum/mesencephalon of *Ctsk*^-/- ^mice, may therefore carry a multitude of consequences, including an overall enhancement of neuronal activity and/or metabolism. Moreover, the decrease in the activity levels of cathepsin B correlated with a strong increase in the levels of synaptotagmin in the striatum/mesencephalon, suggesting that mechanisms mediating neuronal plasticity may be altered in *Ctsk*^-/- ^mice.

Cathepsin L, the most similar in structure and function to cathepsin K, showed a significant decrease in protein levels in the cortex of *Ctsk*^-/- ^mice, while in the hippocampus an increase was observed. Activity, however, was moderately decreased in the striatum/mesencephalon, hippocampus, and cerebellum. A series of studies suggest that cathepsin L is involved in the maturation of the peptide neurotransmitter enkephalin from its proenkephalin precursor [40]. In turn, enkephalin has been shown to interact with the dopaminergic system via D2 receptors [41].

It is interesting to note that we have observed variations not only in the expression levels of cathepsins B and L, but also in those of cystatin C, an endogenous cathepsin inhibitor with extracellular functions [42]. Cystatin C may act as a safe-guarding inhibitor of cysteine cathepsins after their release from damaged cells under challenging conditions that involve cell death [43]. A recent study described cystatin C as having neuroprotective effects on murine primary cortical neurons and neuronal cell lines, by inducing autophagy and thereby improving clearance of long-lived proteins [44]. Cystatin C is also a well established marker of brain tumours such as gliomas [45]. The significance of cystatin C upregulation in the hippocampus of *Ctsk*^-/- ^mice, thus, needs to be further investigated.

Cystatin B, another endogenous cysteine cathepsin inhibitor with mainly intracellular safe-guarding tasks, has been shown previously to be required for normal CNS function. Patients with cystatin B mutations suffer from the Unverricht Lundborg syndrome, a disorder associated with epileptic seizures [46]. Because the cathepsin K-deficient mice exhibited striking neurobehavioral phenotypes, although no signs of epilepsy were detectable, it will be important in the future to determine the levels of cysteine cathepsin inhibitors in the CNS of *Ctsk*^-/- ^mice in more detail. In an elegant approach aiming at the understanding of fine-tuning proteolytic activities at the genome level, mouse models of cystatin B deficiency have been crossed with cathepsin B- and/or L-deficient animals [19] but not yet with *Ctsk*^-/- ^mice. From the results of this study and those of Houseweart and colleagues it is, however, clear that altered ratios of cysteine proteases and their inhibitors occur in the brain of cysteine cathepsin and/or cystatin-deficient mice. This is of relevance, considering that certain inherited mutations or an imbalance of cathepsins and their endogenous inhibitors in the brain of both mice and humans have been linked to a number of severe impairments [18,19,46].

### Changes in cellular architecture resulting from cathepsin K deficiency

In the present work, we observed significantly fewer astrocytes in the hippocampus and significantly more in the cortex of *Ctsk*^-/- ^mice. Astrocytes have a wide range of functions that are highly relevant for CNS homeostasis and for proper neuronal function [47,48]. In pathological situations, including neurodegeneration, activation of astrocytes as characterized by an overexpression of GFAP [49], and astrogliosis, an abnormal increase in the number of astrocytes in damaged areas of the CNS, has been observed [48]. It is therefore not surprising that due to changes in maturation and numbers of astrocytes in *Ctsk*^-/- ^mice, other cell types like oligodendrocytes and neurons may be affected as well. An enhanced signal of the microglial marker Iba1 was detected in parallel to the increased astrocyte population in the cortex of *Ctsk*^-/- ^animals. This is interesting to note, because microglia respond to any kind of injury or perturbation of the CNS and it is believed that they play an essential role both in acute neuroinflammation and in wound healing [28,50]. Therefore, higher levels of cortical Iba1 are in line with the observations of higher GFAP levels in the cortex of *Ctsk*^-/- ^mice, thereby putatively hinting to an altered composition of neurosupportive, neurotrophic, and repair-promoting factors in some regions of the brain.

### Dopaminergic system of *Ctsk*^-/- ^mice and impact on neurobehavioral tasks

The overall changes observed in the dopaminergic system of *Ctsk*^-/- ^mice are of particular interest, since dopamine-mediated neuromodulation is involved in a wide variety of processes, including movement and motor learning, novelty seeking, reward processing, fear conditioning, food intake, nociception, and endocrine and autonomic regulation [29,51,52]. In addition, the dopaminergic system is known to have an important role in both spatial and non-spatial learning [53]. *Ctsk*^-/- ^mice in the present study displayed significantly increased levels of tyrosine hydroxylase, the initial and rate-limiting enzyme in dopamine biosynthesis [54] and slightly increased levels of dopamine. Interestingly, D2-receptor levels were also significantly increased in the striatum/mesencephalon fraction of *Ctsk*^-/- ^mice. Agonists of D2 receptors, such as RU 24213 or quinpirole have been shown to induce learning impairments [55] and to decrease anxiety levels in mice [56], respectively. In line with this notion, in the present study we observed that *Ctsk*^-/- ^mice displayed reduced anxiety levels and impairments in learning and memory skills while exhibiting enhanced dopamine and increased D2-receptor levels in comparison to WT controls.

Another mechanism by which cathepsin K might affect the dopaminergic system could be via its contribution to opioid metabolism. Up-regulation of cathepsin K expression in rats was directly linked to schizophrenia induced by treatment with neuroleptics [22]. Interestingly, *post-mortem *brain tissue from patients with schizophrenia also revealed an increase in the cathepsin K protein levels [14]. A recent *in vitro *study has further shown that cathepsin K can metabolize β-endorphin to produce met-enkephalin [23]. Met-enkephalin binds to opiate receptors, leading to an inhibitory effect on dopaminergic neurons of the VTA [57]. Accordingly, we propose that in the absence of cathepsin K activity, less met-enkephalin would be produced, thus lowering the inhibitory effect on the VTA dopaminergic neurons and leading to increased dopaminergic output.

The deregulated cysteine cathepsin network, the alterations in the cellular architecture of the hippocampus, and the marked metabolic changes observed in *Ctsk*^-/- ^mice which included the dopaminergic system, prompted us to investigate whether these modifications resulted in altered behavioral patterns. Since both the hippocampus and the dopaminergic system are crucially involved in learning and memory [51] as well as in anxiety-related behavior [52], we focused on several tests addressing these aspects.

It is important to mention that, although cathepsin K is well known for its implication in bone remodeling [4,7,58], the mice used in this study did not show any sign of physical impairment, likely because they were used at a young age, of up to 6 months. Moreover, a pilot study on a separate group of *Ctsk*^-/- ^and WT animals (n = 10 for each genotype), using the pole test and the wire hang test, which assess motor abilities in mice [59], showed no difference between the two groups [Sîrbulescu, Jordans, Lerchl, Saftig, Brix; unpublished observations].

In a series of tests chosen to assess anxiety, exploration and memory [60-62], *Ctsk*^-/- ^mice showed decreased anxiety and marked spatial and non-spatial learning and memory impairments. Previous studies have linked an overactive dopaminergic system to reduced inhibition in rodents [51,63]. Our results of the behavioral tests are therefore in line with the observed increase of dopaminergic output in *Ctsk*^-/- ^mice. Moreover, the neuronal cytoarchitecture of the ventral hippocampus was affected more severely by cathepsin K deficiency than that of the dorsal hippocampus. The ventral hippocampus has been shown to be involved in regulating emotion-related behavior [64]. Lesions applied in this area typically lead to reduced anxiety levels in rodents [65]. In addition, the alterations in astrocyte numbers and maturation states observed in the ventral hippocampus of *Ctsk*^*-/- *^animals may contribute to the observed changes in the distribution and numbers of neurons, which in turn can lead to the observed behavioral phenotypes.

*Ctsk*^*-/- *^mice also showed marked impairments in both non-spatial learning, as indicated by their inability to discriminate the introduction of a novel object into a familiar environment, and in spatial learning tasks, as demonstrated by the multiple choice maze paradigm. These results are in agreement with the profound metabolic disruptions found at the level of both the dopaminergic system and the hippocampus in these animals, because the dopaminergic system is known to have an important role in spatial and non-spatial learning [53] while the hippocampus is well established as a structure with an essential role for spatial but also for non-spatial memory formation [64].

## Conclusion

Our results suggest that cathepsin K deficiency in mice has a multiple-level impact on brain development and metabolism. To learn more about which cysteine cathepsins may be most crucial in substituting for the decreased cathepsin K activity, it will be important to identify the natural substrates of cathepsin K in the CNS. Another approach could include mice with double deficiencies in cathepsins B and K, or K and L. The use of such double-deficient animals has been instrumental in specifying the thyroid functions of cathepsin K and helped identifying thyroglobulin as one of its natural targets [1,8,12,13,16].

From this and other recent studies it becomes clear at this point that the activity of cathepsin K has far-reaching effects throughout various organs. This is of highest importance in light of recent advances in the development of targeted cathepsin K inhibitors, aimed at treating osteoporosis [8,20,58]. Such compounds are already in late clinical trials and may be used in patients in the near future [7,34]. In particular, basic cathepsin K inhibitors such as Balicatib and L-006235 have been reported to increase the expression and activity levels of cathepsins B and L in peripheral organs of rats, i.e. liver, kidney, and spleen, but also in the CNS, by factors reaching up to 80-fold [6]. Therefore, clinical trials are now focused on non-basic cathepsin K inhibitors such as Odanacatib, which is currently in phase III clinical trials and expected to be completed in 2012 [34,58]. It has been pointed out, however, that long-term safety and implications of inhibition of cathepsin K activity are not fully known at this point [7,8,58].

Interestingly, pycnodysostosis patients suffering from lifelong absence of cathepsin K activity have been occasionally reported to exhibit, in addition to the abnormalities in bone development, alterations in the CNS such as hyperplasia of the pituitary, demyelination of the cerebrum, and imbalances between brain growth, vascular supply, and cerebrospinal fluid pressure, with complications including sensorineural deafness and frontal porencephalic cysts [66].

We suggest that *Ctsk*^-/- ^mice provide a useful tool to better understand the multiple functions that cathepsin K fulfils in the CNS, in addition to its well-established roles in peripheral tissues. Considering the above-mentioned observations in mice, rats, and humans, it will be worthwhile to address off-target effects of cathepsin K inhibitors on the CNS of patients, since a dopaminergic induction similar to the one observed in this study could help in the treatment of neurodegenerative disorders, such as Parkinson's disease, in addition to managing the symptoms of osteoporosis.

## Methods

### Animals

All studies were performed on 12-26 weeks-old male *Ctsk*^-/- ^or WT C57Bl/6J mice. The mice were back-crossed to a congenic C57Bl/6J background in the animal facility of Jacobs University Bremen, Germany. Back-crossing was performed over 6 generations for mice used in the investigations of the brain cytoarchitecture, and in the studies of metabolic markers. Mice back-crossed over 8 to 11 generations were used for PCR, cathepsin immunofluorescence and activity studies. Generation of the founder *Ctsk*^-/- ^mice at University of Göttingen, Germany, and the genotyping methods are described elsewhere [4]. For the experiments, mice were singly housed under standard conditions, with a 12 h/12 h light/dark cycle with lights out at 07:00 p.m. and *ad libitum *water and food. Testing was conducted in accordance with institutional guidelines, in S1-laboratories of Jacobs University Bremen (SfAFGJS Az. 513-30-00/2-15-32 and 522-27-11/3-1, 05-A20 and A21).

### Activity frequency recordings

Analysis of diurnal variations in locomotor activity was performed as described [67] using 4-6 months old, male *Ctsk*^-/- ^mice and WT controls (n = 5 per genotype). Each mouse was singly-housed over a time period of four weeks, and the activity patterns were determined for the last three weeks. Movements were measured via passive infrared sensor detectors (PIDs; 35 × 29.5 × 20 mm; L × W × H) (Conrad Electronic SE, Hirschau, Germany) which were installed with a coverage angle of 100° about 13 cm above each cage. A QuickBasic program was written for the registration of locomotor activity by PIDs (by courtesy of Dr. Steinlechner, Tierärztliche Hochschule Hannover, Germany) checking all PID channels and the channel for the light sensor.

### Behavioral testing

Behavioral tests were carried out during the light phase, between 2 and 5 p.m. The order of testing for the two genotypes was routinely alternated in order to reduce effects of diurnal rhythm. Behavioral tests were videotaped using a webcam positioned directly above the testing area and visualized on a computer screen. Behavior was coded directly, using Ethograph 2.06 software (Ritec, St. Petersburg, Russia).

#### Elevated plus maze

The plus-shaped maze was constructed from opaque white Plexiglas^®^, with 4 arms of equal length (50 cm) and width (5 cm) extending from a central square platform (side: 5 cm). Closed arms were bordered by side walls (height: 20 cm), while the two open arms did not have any lateral boundaries. The maze was elevated on poles 50 cm above ground. Each mouse was placed on the central platform, facing the same closed arm, and allowed to explore the maze for a single trial lasting 5 min, after which it was returned to its home cage. The time spent in closed or open arms as well as on the central platform was recorded for each individual.

#### Hole board test

A square wooden box of 60 × 60 × 35 cm was used for testing. The inside of the box was painted black, and the floor was divided into nine equal squares. Four holes with a diameter of 3.5 cm were located at the corners of the central square. The floor was elevated 10 cm above the bottom of the box. Individual animals were placed in the lower left corner of the enclosure, facing the wall, and allowed to explore freely for 3 min, after which the mice were returned to the home cage. The number of head dips into holes, rearings, and squares crossed were recorded as described above, the latter serving as a general index of locomotor activity. The number of center crossings was recorded and considered as an indirect measure of anxiety.

#### Novel object recognition test

A box similar to the one described above was used, however lacking the floor holes. Four different objects were placed in squares 1, 4, 6 and 8 during the test: a wooden cube (side: 3.5 cm), a plastic Lego^® ^corner (side: 2 cm, height: 1.5 cm), a plastic Lego^® ^house shape (length: 4 cm, width: 2 cm, height: 3 cm) and a gyps ellipsoid (long radius: 5 cm; short radii: 3.5 cm). Additionally, for the object substitution test, a shell (length: 5 cm, width: 2.5 cm, height: 2 cm) was used as a novel object. Mice were placed in the testing arena, as described above. During the first day of testing, the animals were given two consecutive 3-min habituation sessions, with an inter-session interval of one hour. On the next day, one of the objects (cube) was replaced with an unfamiliar one (shell). Mice were again allowed to explore the setting for 3 min. The total time spent in each of the squares containing an object was determined.

#### Elevated multiple-choice maze

The maze was constructed out of opaque white Plexiglas^® ^strips, with a width of 5 cm and a total length of 265 cm, elevated on poles with a height of 50 cm. Animals were placed in the starting segment of the maze, and had to make three correct decisions in order to reach the exit point. There they could descend via a wire net into a cage containing a house, which was placed on the floor and was not readily visible from the maze. The maximum duration of one trial was 3 min. Testing was conducted daily, over an interval of 12 days. An entry into a maze segment that was outside the correct route (0-1-4-7-E-F in this order, see Figure 8C) was counted as a reference memory error, while backtracking into any already visited segment within a given session was counted as a working memory error.

### Tissue sampling

Mice were anesthetized, the abdominal and thoracic cavities were opened and the abdominal aorta was cut. Perfusion was carried out through the heart with 0.9% NaCl supplemented with 200 IU heparin (Braun Melsungen AG, Melsungen, Germany). The head and neck regions were immediately separated and placed on ice.

#### Brain samples

The brain was removed and the two hemispheres were separated. One hemisphere was placed in 4% PFA in 200 mM HEPES, pH 7.4, and left overnight at 4°C, then cryo-preservation was carried out by incubating in 0.5 M and afterwards in 1 M sucrose solution in phosphate-buffered saline (PBS), pH 7.4, for each another 24 h at 4°C. Tissue samples were embedded in Tissue Freezing Medium (Jung, through Leica Microsystems) and stored at -20°C. Alternatively, tissue was frozen using the liquid nitrogen-isopentane method and stored at -80°C until sectioning. The other hemisphere was immediately dissected on ice and the cortex, hippocampus, cerebellum, and striatum together with the mesencephalon and diencephalon (referred to as striatum/mesencephalon fraction) were separated and snap-frozen in liquid nitrogen.

### SDS-PAGE, immunoblotting, immunodetection and densitometry

#### Protein extraction

Tissue samples were homogenized in ice-cold PBS containing 0.5% Triton X-100, using a Potter S homogenizer (Sartorius, Göttingen, Germany) at 1,000 rpm for 5 min. Further extraction was performed by placing the resulting homogenates in a rotary mixer for 40 min at 4°C. After centrifugation at 10,000 ×-*g *and 4°C for 10 min, the supernatants were removed and stored at -20°C.

#### Gel electrophoresis

The protein content of all samples was determined using the Neuhoff assay [68]. Tissue lysates were normalized to equal amounts of protein, boiled in sample buffer and separated on 12.5% acrylamide gels.

#### Immunoblotting and immunodetection

Separated proteins were semi-dry blotted onto nitrocellulose membrane. Blocking was done overnight at 4°C using 5% milk powder in PBS containing 0.3% Tween-20 (PBST). Primary antibodies, sheep anti-tyrosine hydroxylase (AB1542, Chemicon, Billerica, MA, USA), rabbit anti-dopamine D2 receptor (ab21218, Abcam, Cambridge, UK), mouse anti-GFAP (G3893; Sigma-Aldrich Chemie GmbH, Taufkirchen, Germany), mouse anti-CNPase (C5922; Sigma), chicken anti-synaptotagmin (AB9356; Chemicon), rabbit anti-cystatin C (9300; kind gift of Dr. Magnus Abrahamson, Lund, Sweden), goat anti-mouse cathepsin B (GT15047, Neuromics, through Acris Antibodies, Herford, Germany), rabbit anti-human cathepsin D (IM16, Calbiochem-Novabiochem GmbH, Bad Soden, Germany), and goat anti-mouse cathepsin L (GT15049, Neuromics) were applied for 1.5 h at room temperature. The appropriate secondary antibodies, either HRP-conjugated donkey anti-sheep IgG (713-035-003, Immunoresearch Europe Ltd., Suffolk, UK), goat anti-rabbit IgG (4050-05, Southern Biotech, Birmingham, Alabama, USA), rabbit anti-goat IgG (6160-05; Southern Biotech),or goat anti-chicken IgG (6100-05; Southern Biotech) were applied for 1 h at room temperature. As a loading control, membranes were stripped and re-probed for either glyceraldehyde 3-phosphate dehydrogenase (GAPDH), using a rabbit anti-GAPDH primary antibody (ab9485, Abcam), or for β-tubulin, using a rabbit anti-β-tubulin primary antibody (T-3526, Sigma), and an HRP-conjugated goat anti-rabbit secondary antibody, as described above. Immunoreactions were visualized by enhanced chemiluminescence on CL-XPosure film (Pierce through Perbio Science Europe, Bonn, Germany) or on Hyperfilm MP (GE Healthcare Europe GmbH, Munich, Germany).

#### Densitometry

Films from two to seven different blots for each immunodetection and each animal were scanned with a transmitted-light scanner (Desk Scan II version 2.9; Hewlett-Packard Co., Palo Alto, California, USA) and evaluated densitometrically using TINA version 2.09d (Raytest Isotopen-Messgeräte GmbH, Straubenhardt, Germany). The measured optical density/mm^2 ^values, after background subtraction, were normalized within each blot by dividing individual values to the average of the WT mice values in the respective blot. For normalization β-tubulin was used. For averaging over different immunoblot intensities varying from membrane to membrane values are given as percent of control, e.g. the average of the WT values were set to 100% and each single value (WT and *Ctsk*^-/-^) was then compared to this average value. For quantification of cathepsin protein levels (see, Additional file 1) the bands representing the mature forms, i.e. single chain, or heavy and light chains of two-chain-forms of the respective cathepsins were analyzed by densitometry (see also, Additional file 2).

### PCR

Brain tissue from WT (cortex, striatum/mesencephalon, hippocampus and cerebellum) and *Ctsk*^-/- ^(cortex) mice was homogenized in PBS containing 0.5% Triton X-100 as described above. After centrifugation at 12,000 rpm, pellets were collected and used for RNA extraction using Trizol (Invitrogen), chloroform and isopropanol as described in [69]. RNA concentration was determined by NanoDrop™ (Kiesker, Steinfurt, Germany). Reverse transcription was performed using 2U reverse transcriptase (Quiagen, Hilden, Germany) and 1 μg RNA according to the manufacturer's instructions. The following primers were used for amplification: Cathepsin K 5'-GCC AGG ATG AAA GTT GTA TG-3' (forward), 5'-CAG GCG TTG TTC TTA TTC C-3' (reverse); β-actin: 5'-GCC AGG ATG AAA GTT GTA TG-3' (forward), 5'-CAG GCG TTG TTC TTA TTC C-3' (reverse). Cathepsin K cDNA was amplified by an initial denaturation of 1 min at 95°C, followed by 35 cycles, each consisting of 1 min at 95°C (denaturation), 1 min at 53.4°C (annealing), and 1 min at 72°C (elongation). The amplification of β-actin cDNA consisted of 26 repeats of 30-sec cycles of denaturation at 95°C, annealing for 20 sec at 58°C and elongation for 45 sec at 72°C. The reactions were terminated through incubation for 7 min at 72°C.

### ELISA

For determining levels of dopamine, commercially available ELISA kits were used. Tissue extracts from fractions including the dopaminergic system were first acidified by adding HCl to a final concentration of 0.01 N and then used in a dopamine enzyme immunoassay (Dopamine Research EIA, KAPL10-5300, BioSource Europe S.A., Nivelles, Belgium) according to the manufacturer's manual.

### Cathepsin activity assays

#### Cysteine cathepsins

The enzyme activity assay was performed as described in [70] and with variations as detailed in [71] by monitoring cleavage of 5 μM cathepsin B substrate N-benzyloxycarbonyl-arginyl-arginine-7-amido-4-methylcoumarin (Z-Arg-Arg-AMC; Bachem Distribution Services GmbH, Weil am Rhein, Germany) at pH 6.0 for 10 min at 40°C, 1.5 μM CA-074 cathepsin B-specific inhibitor (Merck Biosciences GmbH, Darmstadt, Germany) plus 5 μM cathepsin L substrate N-benzyloxycarbonyl-phenylalanyl-arginine-7-amido-4-methylcoumarin (Z-Phe-Arg-AMC; Bachem Distribution Services GmbH) at pH 5.5 for 10 min at 30°C, and 5.6 μM cathepsin K substrate *ο*-aminobenzoic acid-histidyl-prolyl-glycyl-glycyl-prolyl-glutaminyl- N-(2,4-dinitrophenyl)-ethylene diamine (Abz-HPGGPQ-EDN_2_ph) [12,24] at pH 5.5 for 10 min at 30°C. Negative controls were run in parallel with the addition of 10 μM of the cysteine protease inhibitor E64. Reactions were terminated by adding stop solutions and fluorescence was measured with a Tecan GENios Reader (Tecan Deutschland GmbH, Crailsheim, Germany), using an excitation wavelength of 360 nm and reading the emission at 465 nm for cathepsin B and L activity assays, whereas cathepsin K activity was determined with an excitation at 320 nm and emission set to 420 nm.

#### Cathepsin D

Activity was tested as previously described [72] with variations as detailed in [71] by monitoring cleavage of cathepsin D substrate 7-methoxycoumarin-4-yl-acetyl-glycyl-lysyl-prolyl-isoleucyl-phenylalanyl-phenylalanyl-arginyl-leucyl-lysine (2, 4-dinitrophenyl)-*D*-arginine (MOCAc-Gly-Lys-Pro-Ile-Leu-Phe ~ Phe-Arg-Leu-Lys (Dnp)-*D*-Arg-NH_2_; Merck Biosciences GmbH) at pH 4.0 for 10 min at 40°C. Controls were incubated in addition with 1 μM pepstatin A (Sigma-Aldrich Chemie GmbH, Taufkirchen, Germany). Reactions were stopped and fluorescence was measured using an excitation wavelength of 328 nm and reading the emission at 393 nm.

#### Analysis

All assays were performed in duplicates and repeated 2-4 times. For each genotype, three to six different mice were used, except for cathepsin B and L striatum/mesencephalon, where eight mice were used. Relative fluorescence units (RFU) of controls were subtracted from the values of the corresponding samples. In order to allow averaging over several assays, RFU values were normalized by the averages of the WT measurements within a given assay. For calculation of specific cathepsin K activity, RFU values were divided by protein contents of the respective samples.

### Immuno- and enzyme histochemistry

Brain hemispheres were cut horizontally on a cryostat (Leica CM1900, Leica Microsystems) into 16 μm sections and thaw-mounted serially on microscope slides. Residual embedding material was washed out prior to the staining procedure by incubating slides overnight in PBS at 4°C. Blocking of non-specific binding sites on sections was performed at room temperature by a 4 h- or 1.5 h-incubation with 3% bovine serum albumin (Albumin Fraction V, Roth, Karlsruhe, Germany) including 0.3% or 0.1% Triton X-100 in calcium and magnesium-free PBS (CMF-PBS), respectively. Primary antibodies, mouse anti-tyrosine hydroxylase (MAB318, Chemicon), mouse anti-GFAP (G3893; Sigma), mouse anti-Neuronal Nuclei (NeuN) (MAB377, Millipore, CA, USA), mouse anti-human cathepsin K (IM55, Calbiochem-Novabiochem GmbH), and rabbit anti-Iba1 (016-20001; Wako Pure Chemical Industries, Japan) antibodies were applied overnight at 4°C. Secondary antibodies, goat anti-mouse IgG conjugated with Alexa 546 (A11018, Invitrogen through Molecular Probes, Karlsruhe, Germany) or Alexa 488 (A11017, Invitrogen), and goat anti-rabbit IgG conjugated with Alexa 488 (A11034, Invitrogen) were applied for 4 h on brain sections at room temperature in a moisturized chamber.

#### Cathepsin K activity assays by enzyme histochemistry

Cryo-sections of 0.5% PFA fixed brain tissue were washed several times in PBS to remove embedding medium before pre-incubation for 5 min at 37°C in reducing reaction buffer consisting of 0.2 M ammonium acetate, 0.125 mM β-mercaptoethanol, 0.1 mM EDTA-Na_2_, 0.5 M Na_2_HPO_4_, pH 6.2, and subsequent incubation under oxygen-free conditions in reaction buffer supplemented with 1 mM cathepsin K substrate Z-glycinyl-prolyl-arginyl-4-methoxy-β-naphthylamine (Z-Gly-Pro-Arg-4MβNA; J1105, Bachem) for 45 min in the presence of 1 mM nitrosalicyl aldehyde (NSA) as the precipitating agent of released 4MβNA as previously described [12]. Thereafter, sections were washed and mounted in PBS, and the insoluble precipitates were visualized at an excitation of 435 - 490 and the emission set to 520 - 530 nm by META-detection with the LSM 510 (see below).

### Microscopy and analysis

Confocal images were taken using a Zeiss LSM 510 META laser-scanning microscope equipped with Argon and Helium-Neon lasers (Carl Zeiss GmbH, Oberkochen, Germany). Optical sections were taken with a pinhole opening of 1 Airy unit and at a resolution of 1,024 × 1,024 pixels, using LSM 5 software (version 3.2; Carl Zeiss).

#### Analysis of hippocampus sections

A total of 278 horizontal sections from the ventral hippocampus and 582 horizontal sections of the dorsal hippocampus were immunolabeled for NeuN and analyzed. For each genotype, six different mice were used. Confocal images taken from the DG, CA1, CA2, and CA3 regions were analyzed using CellProfiler 2.0, version 9777 [73]. The image processing and analysis command pipelines were run under MATLAB (The MathWorks, Inc., Natick, MA). Briefly, images were exported as greyscale pictures and neuronal nuclei were identified according to intensity by application of thresholding and smoothing filters. The number of nuclei per image and the number of nearest neighbours within a 15-pixel (~7 μm) distance of any given nucleus were determined. In order to take into account that neuronal numbers may differ, special care was taken in order to analyze comparable numbers of neurons.

### Statistical analysis

Normal distribution of all data sets was verified using the Kolmogorov-Smirnov test. Two-tailed Student's t-test was used to assess group differences for data from the elevated plus maze, hole board test, immunoblot densitometry, ELISA and morphometric analyses. For analyzing data from the novel object recognition test and elevated multiple choice maze, repeated measures analysis of variance (ANOVA) was used, with significance levels for multiple comparisons corrected using the Bonferroni procedure. For assessing differences in the neuronal distributions the Kolmogorov-Smirnov and the Mann-Whitney U test were used. All data are shown as mean ± standard error of the mean, except for Figure 2 and Additional file 3, which show mean ± standard deviation.

## List of abbreviations

4MβNA: 4-methoxy-β-naphtylamine; Abz-HPGGPQ-EDN_2_pH - *ο*-aminobenzoic acid-histidyl-prolyl-glycyl-glycyl-prolyl-glutaminyl-N-(2,4-dinitrophenyl)-ethylene diamine; bps: basepairs; CathB: cathepsin B; CathD: cathepsin D; CathK: cathepsin K; CathL: cathepsin L; CysC: cystatin C; Cb: cerebellum; CNPase: cyclic-nucleotide-phosphodiesterase; CNS: central nervous system; Co: control; *Ctsd*^-/-^: cathepsin D deficient; *Ctsk*^-/-^: cathepsin K deficient; Cx: cerebral cortex; D2R: dopamine type 2 receptors; DG: dentate gyrus; ELISA: enzyme linked immunosorbent assay; GFAP: glial fibrillaric acidic protein; GV: grey value; H: hippocampus; Iba1: ionized calcium binding adapter protein 1; NeuN: neuronal nuclei; NF-M: neurofilament medium; NSA: nitrosalicylaldehyde; PID: passive infrared sensor detectors; RT-PCR: reverse transcriptase - polymerase chain reaction; RM: reference memory; St: standard; Str or striatum/mesenceph. - striatum/mesencephalon; THase: tyrosine hydroxylase; VTA: ventral tegmental area; WM: working memory; WT: wild type; Z-GPR-4MβNA: Benzyloxycarbonyl-glycyl-prolyl-arginyl-4-methoxy-β-naphthylamine

## Competing interests

The authors declare that they have no competing interests.

## Authors' contributions

SD contributed to the design of the study, carried out the experiments described in Figures 1A and 1B, 2C - 2F, 4, 5A - 5D, 6, 7, and Additional files 1, 2, and 3, and participated in manuscript writing. RFS contributed to the initial concept and design of the study, carried out the experiments displayed in Figures 5E - 5O, 8, 9, and additional file 4, performed statistical analyses for the neuronal distribution and participated in manuscript writing. SJ supervised the initial studies, contributed to manuscript drafting and discussed the data. MR helped with immunofluorescence and immunoblotting experiments, was involved in analyses depicted in Figures 1C - 1F, 2A and 2B, 5E - 5O and 9C. LA carried out investigations shown in Figure 3F - 3G while JO was involved in the respective analysis described in Figure 3A - 3E. AL helped in designing and carrying out the behavioral experiments, provided materials for the experiments described in Figure 8 and additional files 3 and 4, and discussed the data. PS provided the *Ctsk*^-/- ^mice, discussed the data and participated in manuscript drafting. KBr conceived and coordinated the study, participated in its design, supervised the experiments, contributed to data interpretation and participated in manuscript writing. All authors read and approved the final version of the manuscript.

## Supplementary Material

Additional file 1 **Proteolytic network in specific brain regions of *Ctsk*^-/- ^mice as compared to WT controls**. Densitometry analysis of immunoblots and results of cathepsin activity assays performed with brain tissue lysates of WT (open bars) and *Ctsk*^-/- ^mice (grey bars) **(A-D, A'-D') **Cathepsin D protein levels were unaltered and its activity as determined by cleavage of MOCAc-Gly-Lys-Pro-Ile-Leu-Phe ~ Phe-Arg-Leu-Lys (Dnp)-*D*-Arg-NH_2 _at pH 4.0 was slightly elevated in cerebellum of *Ctsk*^-/- ^mice, but these changes were not significant (A, *Ctsk*^-/- ^n = 20, WT n = 19; A', *Ctsk*^-/- ^n = 5, WT n = 5; B, *Ctsk*^-/- ^n = 15, WT n = 17; B', *Ctsk*^-/- ^n = 5, WT n = 5; C, *Ctsk*^-/- ^n = 16, WT n = 17; C', *Ctsk*^-/- ^n = 5, WT n = 5; D, *Ctsk*^-/- ^n = 8, WT n = 12; D', *Ctsk*^-/- ^n = 5, WT n = 5). **(E-H, E'-H') **Cathepsin B protein levels were decreased in cerebral cortex of *Ctsk*^-/- ^mice, while its activity as determined by cleavage of Z-Arg-Arg-AMC at pH 6.0 was reduced only in striatum/mesencephalon of these mice (E, *Ctsk*^-/- ^n = 18, WT n = 19; E', *Ctsk*^-/- ^n = 5, WT n = 5; F, *Ctsk*^-/- ^n = 14, WT n = 15; F', *Ctsk*^-/- ^n = 8, WT n = 8; G, *Ctsk*^-/- ^n = 16, WT n = 18; G', *Ctsk*^-/- ^n = 5, WT n = 5; H, *Ctsk*^-/- ^n = 13, WT n = 17; H', *Ctsk*^-/- ^n = 5, WT n = 5). **(I-L, I'-L') **Cathepsin L protein levels were down-regulated in cerebral cortex, while its Z-Phe-Arg-AMC cleaving activity at pH 5.5 was decreased in striatum/mesencephalon (I, *Ctsk*^-/- ^n = 19, WT n = 19; I', *Ctsk*^-/- ^n = 5, WT n = 5; J, *Ctsk*^-/- ^n = 13, WT n = 15; J', *Ctsk*^-/- ^n = 8, WT n = 8; K, *Ctsk*^-/- ^n = 16, WT n = 18; K', *Ctsk*^-/- ^n = 5, WT n = 5; L, *Ctsk*^-/- ^n = 13, WT n = 17; L', *Ctsk*^-/- ^n = 5, WT n = 5). **(M-P) **Protein levels of the endogenous cysteine peptidase inhibitor, cystatin C, were significantly upregulated in striatum/mesencephalon and hippocampus of *Ctsk*^-/- ^mice (M, *Ctsk*^-/- ^n = 15, WT n = 17; N, *Ctsk*^-/- ^n = 14, WT n = 12; O, *Ctsk*^-/- ^n = 14, WT n = 17; P, *Ctsk*^-/- ^n = 17, WT n = 16). The results indicated deregulated cysteine cathepsins B and L and cystatin C levels depending on the particular brain region analyzed in *Ctsk*^-/- ^mice, whereas the neuroprotective aspartic protease cathepsin D remained unaffected. Levels of significance are denoted as * for p < 0.05; ** for p < 0.01; *** for p < 0.001. Click here for file

Additional file 2 **Cathepsin and cystatin C status in the cerebral cortex, striatum/mesencephalon, cerebellum, and hippocampus**. Representative immunoblots for densitometry analysis shown in Additional file 1; lanes represent separate individuals. (**A-D**) Cathepsin D (heavy chain and light chain) expression. (**E-H) **Cathepsin B (single chain) expression. (**I-L**) Cathepsin L (heavy chain) expression. (**M-P**) Cystatin C expression. Corresponding loading controls (β-tubulin) for each immunoblot are shown in the lower panels. Click here for file

Additional file 3 **Locomotor activity analysis by infrared sensor module recordings of activity frequencies**. Mice were singly housed over a time period of four weeks and recordings were taken by integrating activity frequencies over time intervals of 10 min, each, as detected by the infrared sensor module throughout weeks 2-4. *Ctsk*^-/- ^mice (grey dotted line) exhibited no obvious differences in locomotor activity and diurnal rhythm in comparison to WT controls (straight black line) (*Ctsk*^-/- ^n = 5; WT n = 5). Click here for file

Additional file 4 **Elevated plus maze and hole board test**. (A) *Ctsk*^-/- ^mice (grey bars) spent approximately 36% of the total time in the open arms, a marked increase compared to the WT controls (open bars). In addition, while the WT mice showed a pronounced preference for the closed arms of the maze, this difference was not significant in *Ctsk*^-/- ^mice. **(B) **The hole board test revealed a significant increase in the frequency of central area crossing in *Ctsk*^-/- ^mice compared to WT controls (A and B, *Ctsk*^-/- ^n = 10; WT n = 12). Levels of significance are denoted as * for p < 0.05; *** for p < 0.001. Click here for file

## References

[B1] Brix K Dunkhorst A Mayer K Jordans S Cysteine cathepsins: cellular roadmap to different functions Biochimie 2008 90 194 207 10.1016/j.biochi.2007.07.024 17825974

[B2] Bromme D Okamoto K Human cathepsin O2, a novel cysteine protease highly expressed in osteoclastomas and ovary molecular cloning, sequencing and tissue distribution Biol Chem Hoppe Seyler 1995 376 379 384 10.1515/bchm3.1995.376.6.379 7576232

[B3] Gelb BD Shi GP Chapman HA Desnick RJ Pycnodysostosis, a lysosomal disease caused by cathepsin K deficiency Science 1996 273 1236 1238 10.1126/science.273.5279.1236 8703060

[B4] Saftig P Hunziker E Wehmeyer O Jones S Boyde A Rommerskirch W Moritz JD Schu P von Figura K Impaired osteoclastic bone resorption leads to osteopetrosis in cathepsin-K-deficient mice Proc Natl Acad Sci USA 1998 95 13453 13458 10.1073/pnas.95.23.13453 9811821 PMC24840

[B5] Kiviranta R Morko J Uusitalo H Aro HT Vuorio E Rantakokko J Accelerated turnover of metaphyseal trabecular bone in mice overexpressing cathepsin K J Bone Miner Res 2001 16 1444 1452 10.1359/jbmr.2001.16.8.1444 11499867

[B6] Desmarais S Masse F Percival MD Pharmacological inhibitors to identify roles of cathepsin K in cell-based studies: a comparison of available tools Biol Chem 2009 390 941 948 10.1515/BC.2009.092 19453281

[B7] Podgorski I Future of anticathepsin K drugs: dual therapy for skeletal disease and atherosclerosis? Future Med Chem 2009 1 21 34 10.4155/fmc.09.4 20126511 PMC2780340

[B8] Dauth S Arampatzidou M Rehders M Yu DMT Führer D Brix K Thyroid cathepsin K - roles in physiology and thyroid disease Clin Rev Bone and Miner Metab 2011 9 94 106 10.1007/s12018-011-9093-7

[B9] Punturieri A Filippov S Allen E Caras I Murray R Reddy V Weiss SJ Regulation of elastinolytic cysteine proteinase activity in normal and cathepsin K-deficient human macrophages J Exp Med 2000 192 789 799 10.1084/jem.192.6.789 10993910 PMC2193285

[B10] Buhling F Gerber A Hackel C Kruger S Kohnlein T Bromme D Reinhold D Ansorge S Welte T Expression of cathepsin K in lung epithelial cells Am J Respir Cell Mol Biol 1999 20 612 619 10100992 10.1165/ajrcmb.20.4.3405

[B11] Haeckel C Krueger S Buehling F Broemme D Franke K Schuetze A Roese I Roessner A Expression of cathepsin K in the human embryo and fetus Dev Dyn 1999 216 89 95 10.1002/(SICI)1097-0177(199910)216:2<89::AID-DVDY1>3.0.CO;2-9 10536050

[B12] Tepel C Bromme D Herzog V Brix K Cathepsin K in thyroid epithelial cells: sequence, localization and possible function in extracellular proteolysis of thyroglobulin J Cell Sci 2000 113 4487 4498 11082042 10.1242/jcs.113.24.4487

[B13] Jordans S Jenko-Kokalj S Kuhl NM Tedelind S Sendt W Bromme D Turk D Brix K Monitoring compartment-specific substrate cleavage by cathepsins B, K, L, and S at physiological pH and redox conditions BMC Biochem 2009 10 23 10.1186/1471-2091-10-23 19772638 PMC2759951

[B14] Bernstein HG Bukowska A Dobrowolny H Bogerts B Lendeckel U Cathepsin K and schizophrenia Synapse 2007 61 252 253 10.1002/syn.20358 17230547

[B15] Sîrbulescu R Jordans S Lerchl A Saftig P Kühl NM Brix K Dolinar M, Stoka V, Turk B and Turk V Trafficking of cysteine cathepsin to the extracellular thyroid follicle lumen helps mice to improve their memory and learning skills In Book of abstracts/Xth International Symposium on Proteinase Inhibitors and Biological Control, Portoroz, Slovenia, June 23-27, 2007 2007 Jožef Stefan Institute, Ljubljana, Slovenia 53

[B16] Friedrichs B Tepel C Reinheckel T Deussing J von Figura K Herzog V Peters C Saftig P Brix K Thyroid functions of mouse cathepsins B, K, and L J Clin Invest 2003 111 1733 1745 12782676 10.1172/JCI15990 PMC156100

[B17] Koike M Nakanishi H Saftig P Ezaki J Isahara K Ohsawa Y Schulz-Schaeffer W Watanabe T Waguri S Kametaka S Shibata M Yamamoto K Kominami E Peters C von Figura K Uchiyama Y Cathepsin D deficiency induces lysosomal storage with ceroid lipofuscin in mouse CNS neurons J Neurosci 2000 20 6898 6906 10995834 10.1523/JNEUROSCI.20-18-06898.2000 PMC6772823

[B18] Felbor U Kessler B Mothes W Goebel HH Ploegh HL Bronson RT Olsen BR Neuronal loss and brain atrophy in mice lacking cathepsins B and L Proc Natl Acad Sci USA 2002 99 7883 7888 10.1073/pnas.112632299 12048238 PMC122989

[B19] Houseweart MK Pennacchio LA Vilaythong A Peters C Noebels JL Myers RM Cathepsin B but not cathepsins L or S contributes to the pathogenesis of Unverricht-Lundborg progressive myoclonus epilepsy (EPM1) J Neurobiol 2003 56 315 327 10.1002/neu.10253 12918016

[B20] Desmarais S Black WC Oballa R Lamontagne S Riendeau D Tawa P Duong le T Pickarski M Percival MD Effect of cathepsin K inhibitor basicity on in vivo off-target activities Mol Pharmacol 2008 73 147 156 17940194 10.1124/mol.107.039511

[B21] Feher LZ Kalman J Puskas LG Gyulveszi G Kitajka K Penke B Palotas M Samarova EI Molnar J Zvara A Matin K Bodi N Hugyecz M Pakaski M Bjelik A Juhasz A Bogats G Janka Z Palotas A Impact of haloperidol and risperidone on gene expression profile in the rat cortex Neurochem Int 2005 47 271 280 10.1016/j.neuint.2005.04.020 15941608

[B22] Ko F Tallerico T Seeman P Antipsychotic pathway genes with expression altered in opposite direction by antipsychotics and amphetamine Synapse 2006 60 141 151 10.1002/syn.20287 16715494

[B23] Lendeckel U Kahne T Ten Have S Bukowska A Wolke C Bogerts B Keilhoff G Bernstein HG Cathepsin K generates enkephalin from beta-endorphin: a new mechanism with possible relevance for schizophrenia Neurochem Int 2009 54 410 417 10.1016/j.neuint.2009.01.011 19428782

[B24] Lecaille F Weidauer E Juliano MA Bromme D Lalmanach G Probing cathepsin K activity with a selective substrate spanning its active site Biochem J 2003 375 307 312 10.1042/BJ20030468 12837132 PMC1223680

[B25] Kingham PJ Pocock JM Microglial secreted cathepsin B induces neuronal apoptosis J Neurochem 2001 76 1475 1484 10.1046/j.1471-4159.2001.00146.x 11238732

[B26] Holscher C Time, space and hippocampal functions Rev Neurosci 2003 14 253 284 10.1515/REVNEURO.2003.14.3.253 14513868

[B27] Asagiri M Hirai T Kunigami T Kamano S Gober HJ Okamoto K Nishikawa K Latz E Golenbock DT Aoki K Ohya K Imai Y Morishita Y Miyazono K Kato S Saftig P Takayanagi H Cathepsin K-dependent toll-like receptor 9 signaling revealed in experimental arthritis Science 2008 319 624 627 10.1126/science.1150110 18239127

[B28] Streit WJ Xue QS Life and death of microglia J Neuroimmune Pharmacol 2009 4 371 379 10.1007/s11481-009-9163-5 19680817

[B29] Vaccari A Rossetti ZL de Montis G Stefanini E Martino E Gessa GL Neonatal hypothyroidism induces striatal dopaminergic dysfunction Neuroscience 1990 35 699 706 10.1016/0306-4522(90)90340-A 2199846

[B30] Kandel ER The biology of memory: a forty-year perspective J Neurosci 2009 29 12748 12756 10.1523/JNEUROSCI.3958-09.2009 19828785 PMC6665299

[B31] Bjorklund A Dunnett SB Dopamine neuron systems in the brain: an update Trends Neurosci 2007 30 194 202 10.1016/j.tins.2007.03.006 17408759

[B32] Rose AA Siegel PM Emerging therapeutic targets in breast cancer bone metastasis Future Oncol 2010 6 55 74 10.2217/fon.09.138 20021209

[B33] Yang M Sun J Zhang T Liu J Zhang J Shi MA Darakhshan F Guerre-Millo M Clement K Gelb BD Dolgnov G Shi GP Deficiency and inhibition of cathepsin K reduce body weight gain and increase glucose metabolism in mice Arterioscler Thromb Vasc Biol 2008 28 2202 2208 10.1161/ATVBAHA.108.172320 18818416 PMC2736686

[B34] Rachner TD Khosla S Hofbauer LC Osteoporosis: now and the future Lancet 2011 377 1276 1287 10.1016/S0140-6736(10)62349-5 21450337 PMC3555696

[B35] Horn S Heuer H Thyroid hormone action during brain development: more questions than answers Mol Cell Endocrinol 2010 315 19 26 10.1016/j.mce.2009.09.008 19765631

[B36] Portella AC Carvalho F Faustino L Wondisford FE Ortiga-Carvalho TM Gomes FC Thyroid hormone receptor beta mutation causes severe impairment of cerebellar development Mol Cell Neurosci 2010 44 68 77 10.1016/j.mcn.2010.02.004 20193766

[B37] Gan L Ye S Chu A Anton K Yi S Vincent VA von Schack D Chin D Murray J Lohr S Patthy L Gonzalez-Zulueta M Nikolich K Urfer R Identification of cathepsin B as a mediator of neuronal death induced by Abeta-activated microglial cells using a functional genomics approach J Biol Chem 2004 279 5565 5572 14612454 10.1074/jbc.M306183200

[B38] Kikuchi H Yamada T Furuya H Doh-ura K Ohyagi Y Iwaki T Kira J Involvement of cathepsin B in the motor neuron degeneration of amyotrophic lateral sclerosis Acta Neuropathol 2003 105 462 468 12677446 10.1007/s00401-002-0667-9

[B39] Graber S Maiti S Halpain S Cathepsin B-like proteolysis and MARCKS degradation in sub-lethal NMDA-induced collapse of dendritic spines Neuropharmacology 2004 47 706 713 10.1016/j.neuropharm.2004.08.004 15458842

[B40] Hook V Yasothornsrikul S Greenbaum D Medzihradszky KF Troutner K Toneff T Bundey R Logrinova A Reinheckel T Peters C Bogyo M Cathepsin L and Arg/Lys aminopeptidase: a distinct prohormone processing pathway for the biosynthesis of peptide neurotransmitters and hormones Biol Chem 2004 385 473 480 10.1515/BC.2004.055 15255178

[B41] Hayward MD Low MJ Naloxone's suppression of spontaneous and food-conditioned locomotor activity is diminished in mice lacking either the dopamine D(2) receptor or enkephalin Brain Res Mol Brain Res 2005 140 91 98 16125819 10.1016/j.molbrainres.2005.07.016

[B42] Abrahamson M Alvarez-Fernandez M Nathanson CM Cystatins Biochem Soc Symp 2003 179 199 10.1042/bss0700179 14587292

[B43] Salvesen G Nagase H Beyon RJ and Bond JS Inhibition of proteolytic enzymes In Proteolytic Enzymes: a Practical Approach 1989 IRL Press at Oxford University 1983 1104

[B44] Tizon B Sahoo S Yu H Gauthier S Kumar AR Mohan P Figliola M Pawlik M Grubb A Uchiyama Y Bandyopadhyay U Cuervo AM Nixon RA Levy E Induction of autophagy by cystatin C: a mechanism that protects murine primary cortical neurons and neuronal cell lines PLoS One 2010 5 e9819 10.1371/journal.pone.0009819 20352108 PMC2843718

[B45] Nakabayashi H Hara M Shimuzu K Clinicopathologic significance of cystatin C expression in gliomas Hum Pathol 2005 36 1008 1015 10.1016/j.humpath.2005.06.021 16153465

[B46] Pennacchio LA Bouley DM Higgins KM Scott MP Noebels JL Myers RM Progressive ataxia, myoclonic epilepsy and cerebellar apoptosis in cystatin B-deficient mice Nat Genet 1998 20 251 258 10.1038/3059 9806543

[B47] Barres BA The mystery and magic of glia: a perspective on their roles in health and disease Neuron 2008 60 430 440 10.1016/j.neuron.2008.10.013 18995817

[B48] Fellin T Communication between neurons and astrocytes: relevance to the modulation of synaptic and network activity J Neurochem 2009 108 533 544 10.1111/j.1471-4159.2008.05830.x 19187090

[B49] Rossi D Volterra A Astrocytic dysfunction: insights on the role in neurodegeneration Brain Res Bull 2009 80 224 232 10.1016/j.brainresbull.2009.07.012 19631259

[B50] Streit WJ Microglia as neuroprotective, immunocompetent cells of the CNS Glia 2002 40 133 139 10.1002/glia.10154 12379901

[B51] Voon V Fernagut PO Wickens J Baunez C Rodriguez M Pavon N Juncos JL Obeso JA Bezard E Chronic dopaminergic stimulation in Parkinson's disease: from dyskinesias to impulse control disorders Lancet Neurol 2009 8 1140 1149 10.1016/S1474-4422(09)70287-X 19909912

[B52] Pezze MA Feldon J Mesolimbic dopaminergic pathways in fear conditioning Prog Neurobiol 2004 74 301 320 10.1016/j.pneurobio.2004.09.004 15582224

[B53] Adriani W Sargolini F Coccurello R Oliverio A Mele A Role of dopaminergic system in reactivity to spatial and non-spatial changes in mice Psychopharmacology (Berl) 2000 150 67 76 10.1007/s002130000423 10867978

[B54] Nagatsu T Levitt M Udenfriend S Tyrosine Hydroxylase. the Initial Step in Norepinephrine Biosynthesis J Biol Chem 1964 239 2910 2917 14216443

[B55] Ichihara K Nabeshima T Kameyama T Effects of dopamine receptor agonists on passive avoidance learning in mice: interaction of dopamine D1 and D2 receptors Eur J Pharmacol 1992 213 243 249 10.1016/0014-2999(92)90688-Z 1355736

[B56] de Oliveira AR Reimer AE Brandao ML Dopamine D2 receptor mechanisms in the expression of conditioned fear Pharmacol Biochem Behav 2006 84 102 111 10.1016/j.pbb.2006.04.012 16780936

[B57] Margolis EB Lock H Chefer VI Shippenberg TS Hjelmstad GO Fields HL Kappa opioids selectively control dopaminergic neurons projecting to the prefrontal cortex Proc Natl Acad Sci USA 2006 103 2938 2942 10.1073/pnas.0511159103 16477003 PMC1413839

[B58] Canalis E New Treatment Modalities in Osteoporosis Endocr Pract 2010 16 855 863 10.4158/EP10048.RA 20350910 PMC3278773

[B59] Karl T Pabst R von Horsten S Behavioral phenotyping of mice in pharmacological and toxicological research Exp Toxicol Pathol 2003 55 69 83 10.1078/0940-2993-00301 12940631

[B60] Rodriguiz RM Wetsel WC Levin ED and Buccafusco JJ Assessments of Cognitive Deficits in Mutant Mice Animal Models of Cognitive Impairment 2006 Boca Raton (FL): CRC Press Chapter 12 21204369

[B61] Pellow S Chopin P File SE Briley M Validation of open:closed arm entries in an elevated plus-maze as a measure of anxiety in the rat J Neurosci Methods 1985 14 149 167 10.1016/0165-0270(85)90031-7 2864480

[B62] Karasawa J Hashimoto K Chaki S D-Serine and a glycine transporter inhibitor improve MK-801-induced cognitive deficits in a novel object recognition test in rats Behav Brain Res 2008 186 78 83 10.1016/j.bbr.2007.07.033 17854919

[B63] Zhuang X Oosting RS Jones SR Gainetdinov RR Miller GW Caron MG Hen R Hyperactivity and impaired response habituation in hyperdopaminergic mice Proc Natl Acad Sci USA 2001 98 1982 1987 10.1073/pnas.98.4.1982 11172062 PMC29368

[B64] Barkus C McHugh SB Sprengel R Seeburg PH Rawlins JN Bannerman DM Hippocampal NMDA receptors and anxiety: at the interface between cognition and emotion Eur J Pharmacol 2010 626 49 56 10.1016/j.ejphar.2009.10.014 19836379 PMC2824088

[B65] Bannerman DM Rawlins JN McHugh SB Deacon RM Yee BK Bast T Zhang WN Pothuizen HH Feldon J Regional dissociations within the hippocampus--memory and anxiety Neurosci Biobehav Rev 2004 28 273 283 10.1016/j.neubiorev.2004.03.004 15225971

[B66] Figueiredo J Reis A Vaz R Leao M Cruz C Porencephalic cyst in pycnodysostosis J Med Genet 1989 26 782 784 10.1136/jmg.26.12.782 2614800 PMC1015763

[B67] Klose M Grote K Lerchl A Temporal control of spermatogenesis is independent of the central circadian pacemaker in Djungarian hamsters (*Phodopus sungorus*) Biol Reprod 2011 84 124 129 10.1095/biolreprod.110.085126 20826727

[B68] Neuhoff V Philipp K Zimmer HG Mesecke S A simple, versatile, sensitive and volume-independent method for quantitative protein determination which is independent of other external influences Hoppe Seylers Z Physiol Chem 1979 360 1657 1670 10.1515/bchm2.1979.360.2.1657 92445

[B69] Chomczynski P Sacchi N Single-step method of RNA isolation by acid guanidinium thiocyanate-phenol-chloroform extraction Anal Biochem 1987 162 156 159 2440339 10.1006/abio.1987.9999

[B70] Barrett AJ Fluorimetric assays for cathepsin B and cathepsin H with methylcoumarylamide substrates Biochem J 1980 187 909 912 6897924 10.1042/bj1870909 PMC1162479

[B71] Mayer K Vreemann A Qu H Brix K Release of endo-lysosomal cathepsins B, D, and L from IEC6 cells in a cell culture model mimicking intestinal manipulation Biol Chem 2009 390 471 480 10.1515/BC.2009.047 19284293

[B72] Yasuda Y Kageyama T Akamine A Shibata M Kominami E Uchiyama Y Yamamoto K Characterization of new fluorogenic substrates for the rapid and sensitive assay of cathepsin E and cathepsin D J Biochem 1999 125 1137 1143 10348917 10.1093/oxfordjournals.jbchem.a022396

[B73] Carpenter AE Jones TR Lamprecht MR Clarke C Kang IH Friman O Guertin DA Chang JH Lindquist RA Moffat J Golland P Sabatini DM CellProfiler: image analysis software for identifying and quantifying cell phenotypes Genome Biol 2006 7 R100 10.1186/gb-2006-7-10-r100 17076895 PMC1794559

