# Supplementary figures and images for: Cathepsin K deficiency in mice induces structural and metabolic changes in the central nervous system that are associated with learning and memory deficits
Source: BMC Neurosci. 2011 Jul 27;12:74. doi: 10.1186/1471-2202-12-74 (PMC3199875; doi:10.1186/1471-2202-12-74)

## Cortex

## Striatum/Mesenceph.

## Cerebellum

## Hippocampus

## CathD

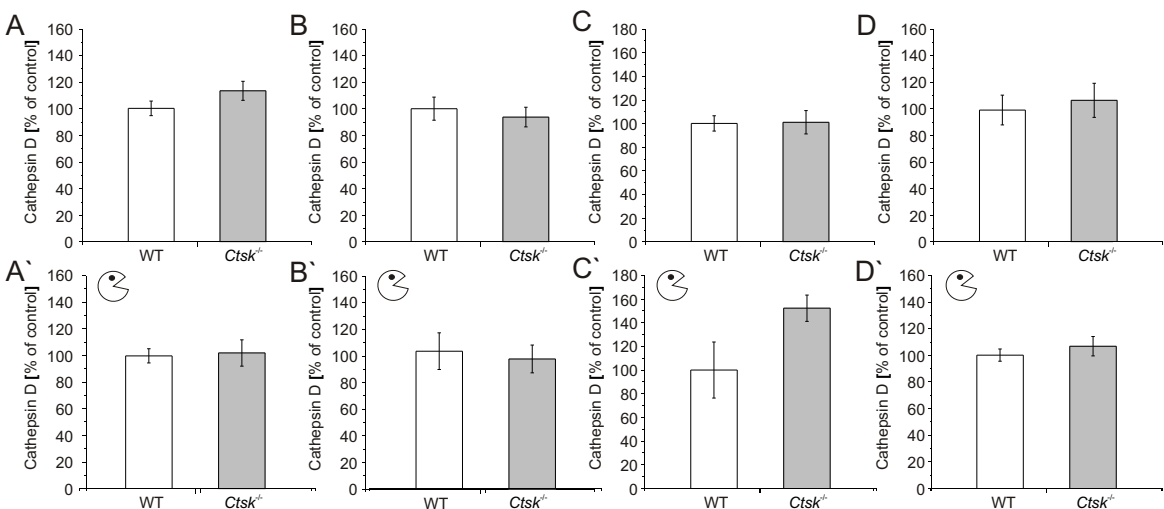

## CathB

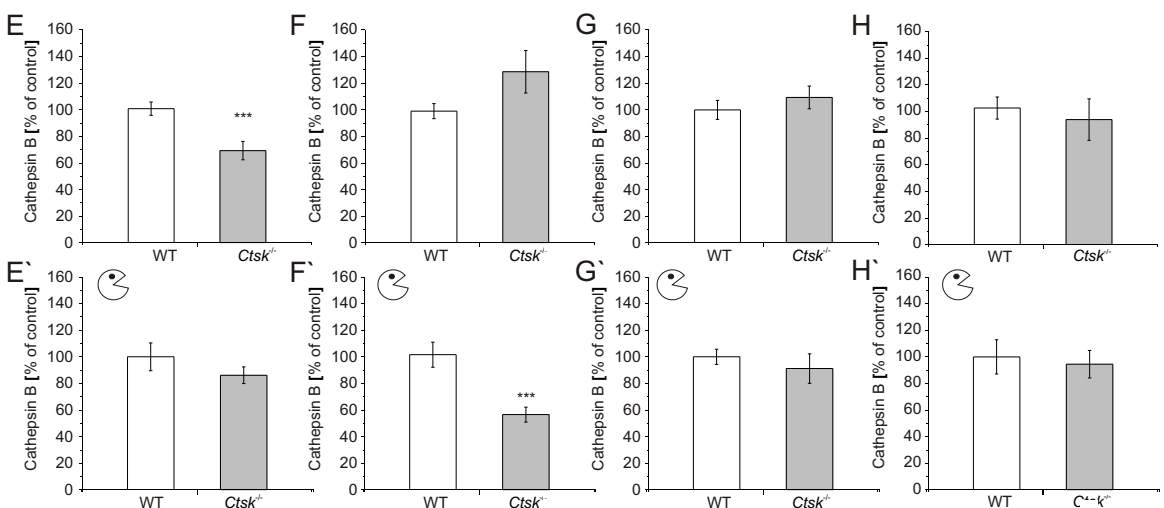

## CathL

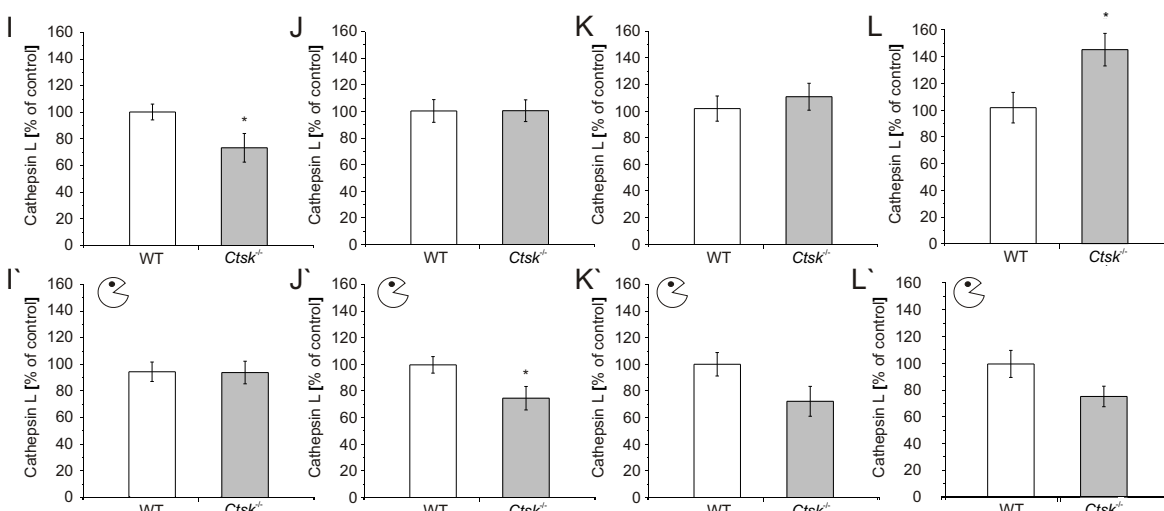

## Cys C

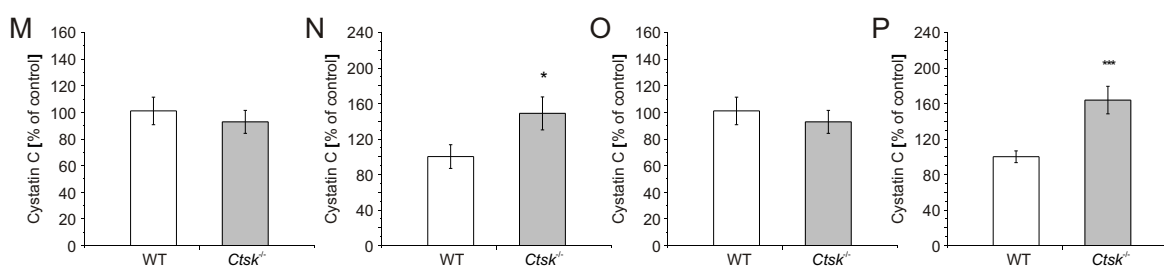

Supplement: Additional file 1 — Proteolytic network in specific brain regions of Ctsk-/- mice as compared to WT controls. Densitometry analysis of immunoblots and results of cathepsin activity assays performed with brain tissue lysates of WT (open bars) and Ctsk-/- mice (grey bars) (A-D, A'-D') Cathepsin D protein levels were unaltered and its activity as determined by cleavage of MOCAc-Gly-Lys-Pro-Ile-Leu-Phe ~ Phe-Arg-Leu-Lys (Dnp)-D-Arg-NH2 at pH 4.0 was slightly elevated in cerebellum of Ctsk-/- mice, but these changes were not significant (A, Ctsk-/- n = 20, WT n = 19; A', Ctsk-/- n = 5, WT n = 5; B, Ctsk-/- n = 15, WT n = 17; B', Ctsk-/- n = 5, WT n = 5; C, Ctsk-/- n = 16, WT n = 17; C', Ctsk-/- n = 5, WT n = 5; D, Ctsk-/- n = 8, WT n = 12; D', Ctsk-/- n = 5, WT n = 5). (E-H, E'-H') Cathepsin B protein levels were decreased in cerebral cortex of Ctsk-/- mice, while its activity as determined by cleavage of Z-Arg-Arg-AMC at pH 6.0 was reduced only in striatum/mesencephalon of these mice (E, Ctsk-/- n = 18, WT n = 19; E', Ctsk-/- n = 5, WT n = 5; F, Ctsk-/- n = 14, WT n = 15; F', Ctsk-/- n = 8, WT n = 8; G, Ctsk-/- n = 16, WT n = 18; G', Ctsk-/- n = 5, WT n = 5; H, Ctsk-/- n = 13, WT n = 17; H', Ctsk-/- n = 5, WT n = 5). (I-L, I'-L') Cathepsin L protein levels were down-regulated in cerebral cortex, while its Z-Phe-Arg-AMC cleaving activity at pH 5.5 was decreased in striatum/mesencephalon (I, Ctsk-/- n = 19, WT n = 19; I', Ctsk-/- n = 5, WT n = 5; J, Ctsk-/- n = 13, WT n = 15; J', Ctsk-/- n = 8, WT n = 8; K, Ctsk-/- n = 16, WT n = 18; K', Ctsk-/- n = 5, WT n = 5; L, Ctsk-/- n = 13, WT n = 17; L', Ctsk-/- n = 5, WT n = 5). (M-P) Protein levels of the endogenous cysteine peptidase inhibitor, cystatin C, were significantly upregulated in striatum/mesencephalon and hippocampus of Ctsk-/- mice (M, Ctsk-/- n = 15, WT n = 17; N, Ctsk-/- n = 14, WT n = 12; O, Ctsk-/- n = 14, WT n = 17; P, Ctsk-/- n = 17, WT n = 16). The results indicated deregulated cysteine cathepsins B and L and cystatin C levels d [file 1471-2202-12-74-S1.PDF]

## Cortex

## Striatum/Mesencephalon

## Cerebellum

## Hippocampus

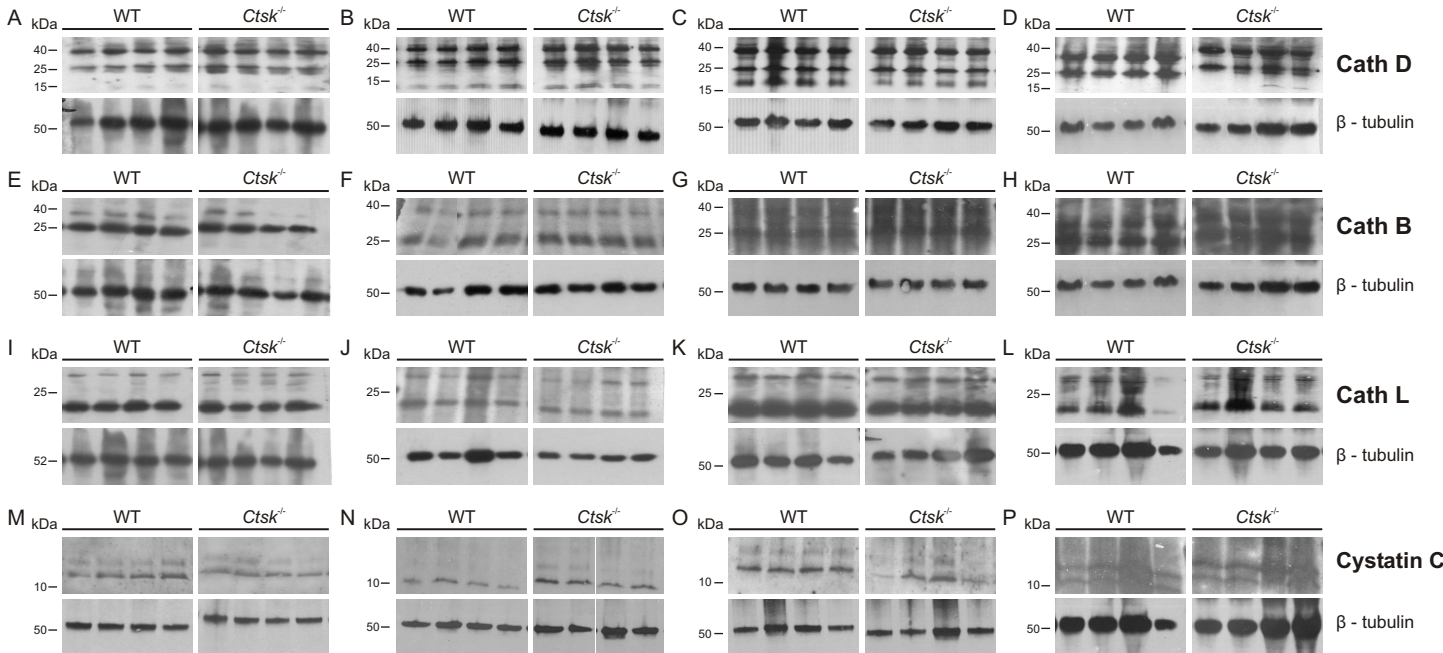

Supplement: Additional file 2 — Cathepsin and cystatin C status in the cerebral cortex, striatum/mesencephalon, cerebellum, and hippocampus. Representative immunoblots for densitometry analysis shown in Additional file 1; lanes represent separate individuals. (A-D) Cathepsin D (heavy chain and light chain) expression. (E-H) Cathepsin B (single chain) expression. (I-L) Cathepsin L (heavy chain) expression. (M-P) Cystatin C expression. Corresponding loading controls (β-tubulin) for each immunoblot are shown in the lower panels. [file 1471-2202-12-74-S2.PDF]

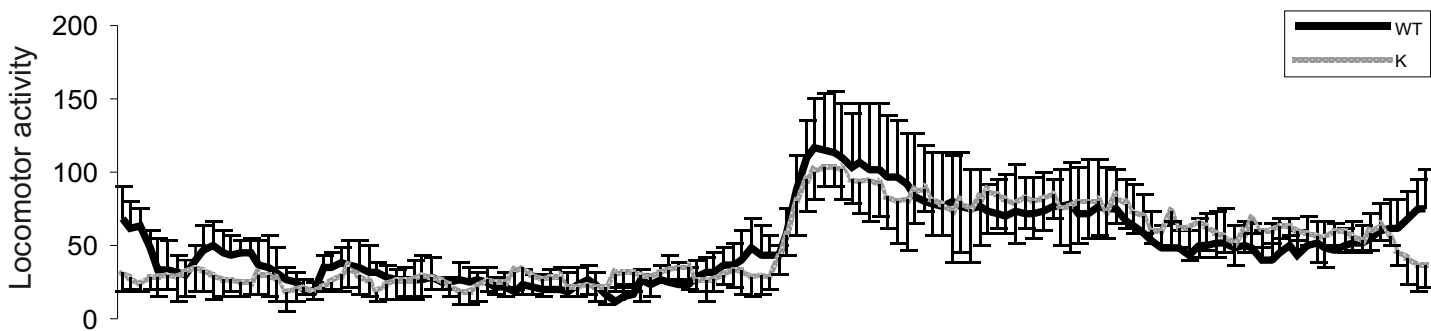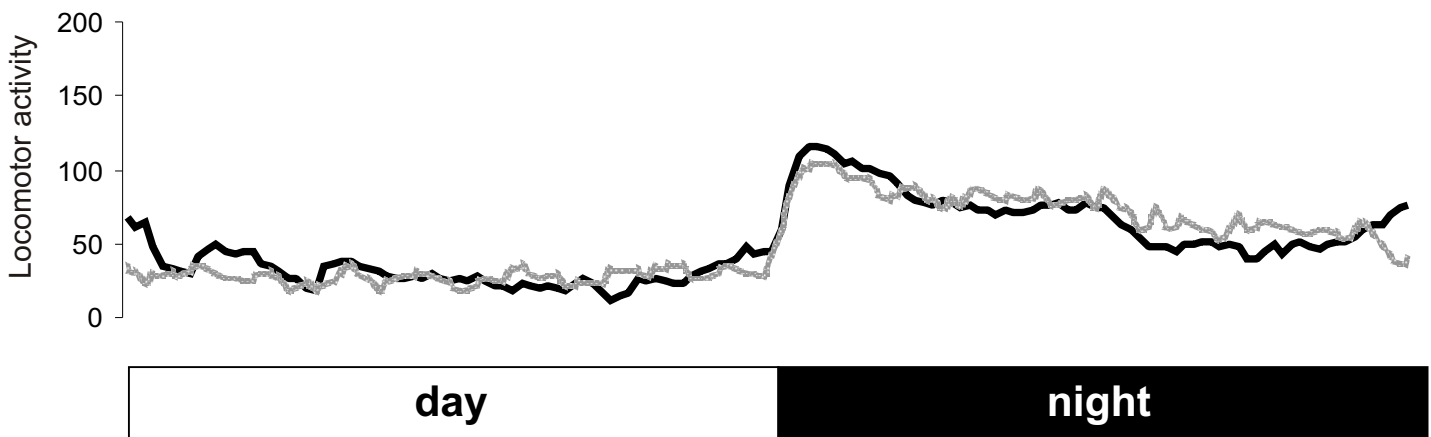

Supplement: Additional file 3 — Locomotor activity analysis by infrared sensor module recordings of activity frequencies. Mice were singly housed over a time period of four weeks and recordings were taken by integrating activity frequencies over time intervals of 10 min, each, as detected by the infrared sensor module throughout weeks 2-4. Ctsk-/- mice (grey dotted line) exhibited no obvious differences in locomotor activity and diurnal rhythm in comparison to WT controls (straight black line) (Ctsk-/- n = 5; WT n = 5). [file 1471-2202-12-74-S3.PDF]

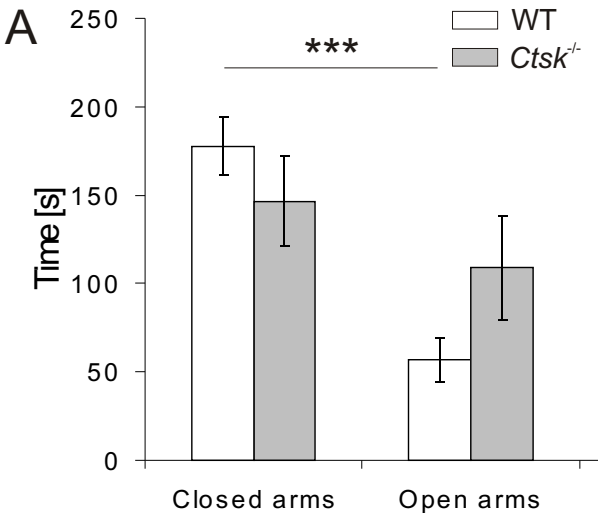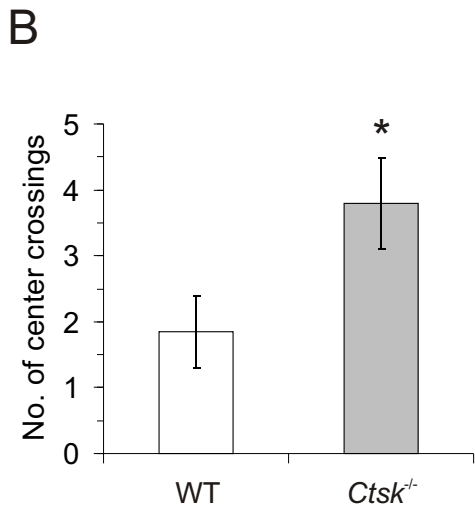

Supplement: Additional file 4 — Elevated plus maze and hole board test. (A) Ctsk-/- mice (grey bars) spent approximately 36% of the total time in the open arms, a marked increase compared to the WT controls (open bars). In addition, while the WT mice showed a pronounced preference for the closed arms of the maze, this difference was not significant in Ctsk-/- mice. (B) The hole board test revealed a significant increase in the frequency of central area crossing in Ctsk-/- mice compared to WT controls (A and B, Ctsk-/- n = 10; WT n = 12). Levels of significance are denoted as * for p < 0.05; *** for p < 0.001. [file 1471-2202-12-74-S4.PDF]
